# Effects of Organic Carbon Origin on Hydrophobic Organic Contaminant Fate in the Baltic Sea

**DOI:** 10.1021/acs.est.1c04601

**Published:** 2021-09-23

**Authors:** Inna Nybom, Gisela Horlitz, Dorothea Gilbert, Naiara Berrojalbiz, Jannik Martens, Hans Peter H. Arp, Anna Sobek

**Affiliations:** † Department of Environmental Science, Stockholm University, 10691 Stockholm, Sweden; ‡ Norwegian Geological Institute (NGI), P.O. Box. 3930, Ullevål Stadion, N-0806 Oslo, Norway; § Department of Environmental Chemistry, IDAEA-CSIC, Jordi Girona 18-26, Barcelona 08034, Catalunya, Spain; ∥ Department of Chemistry, Norwegian University of Science and Technology (NTNU), NO-7491 Trondheim, Norway

**Keywords:** sorption, polycyclic aromatic hydrocarbons (PAHs), polychlorinated biphenyls (PCBs), organic carbon, partitioning, passive sampling, freely dissolved concentration, Baltic Sea

## Abstract

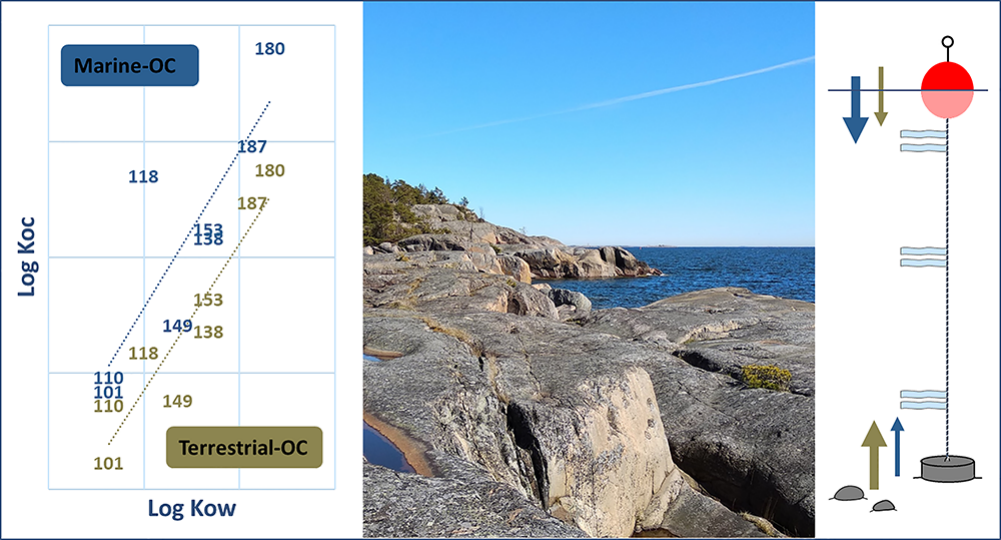

The transport and fate of hydrophobic organic contaminants (HOCs) in the marine environment are closely linked to organic carbon (OC) cycling processes. We investigated the influence of marine versus terrestrial OC origin on HOC fluxes at two Baltic Sea coastal sites with different relative contributions of terrestrial and marine OC. Stronger sorption of the more than four-ring polycyclic aromatic hydrocarbons and penta-heptachlorinated polychlorinated biphenyls (PCBs) was observed at the marine OC-dominated site. The site-specific partition coefficients between sediment OC and water were 0.2–1.0 log units higher at the marine OC site, with the freely dissolved concentrations in the sediment pore-water 2–10 times lower, when compared with the terrestrial OC site. The stronger sorption at the site characterized with marine OC was most evident for the most hydrophobic PCBs, leading to reduced fluxes of these compounds from sediment to water. According to these results, future changes in OC cycling because of climate change, leading to increased input of terrestrial OC to the marine system, can have consequences for the availability and mobility of HOCs in aquatic systems and thereby also for the capacity of sediments to store HOCs.

## Abstract 2

Stronger sorption of contaminants to organic carbon with more marine versus terrestrial origin suggests that climate-change-driven impact on carbon cycling can affect air-to-sediment fluxes of contaminants.

## Introduction

Hydrophobic organic contaminants (HOCs) such as polycyclic aromatic hydrocarbons (PAHs) and polychlorinated biphenyls (PCBs) are ubiquitously present in the aquatic environment.^1−3^ The transport dynamics and ultimate fate of HOCs are dependent on compound properties and diverse environmental processes. For hydrophobic substances, interactions with the organic carbon (OC) cycle are particularly important.^3−6^ Diffusive air–water exchange is a significant source of HOCs to aquatic systems.^4^ In the water column, HOCs partition to mainly natural OC and undergo vertical export with sinking particles to deep-water layers and sediment.^4,5,7^ For example, sorption to the OC of phytoplankton in the photic zone can regulate dissolved water concentrations of HOCs and consequently influence the diffusive exchange between the atmosphere and water and depositional flux of HOCs to sediment.^6^ Sediment is considered a sink for HOCs, particularly in the case of sediment burial,^4,5,8,9^ but on a local scale sediment may act as a secondary source for contaminants when the primary emissions are reduced.^10−13^

Sorption of organic contaminants to naturally occurring terrestrial OC (free from combustion or plastic residues) seems generally less substantial than to naturally occurring marine OC. Previous work demonstrated that the sorption of carbon tetrachloride and 1,2-dichlorobenzene to sediment OC was a factor of 1.8 stronger than to soil OC,^14^ and for PAHs, the effect was a factor of 1.6.^15^ Furthermore, on a local scale, primary production generates marine OC free of organic contaminants in comparison to the terrestrial OC that enters marine systems potentially loaded with organic contaminants from the catchment. As a consequence, a water column with a higher contribution of marine OC would have a greater sorption potential, and therefore a higher affinity to pull contaminants from air to water, and then deposit them from water to sediment, compared to a water body with a higher terrestrial influence, controlling for all other parameters like OC concentration and temperature.^16^ There is, however, also variability in the sorption capacity of various marine OC. For instance, Kuzyk et al.,^17^ observed higher concentrations of PCBs in sediment with a high contribution of marine OC produced under eutrophic conditions compared to oligotrophic conditions. Strong PAH sorption has been observed on aliphatic-rich OC (e.g., algae),^18−20^ and differences in partitioning of HOCs have been observed between different plankton species, size classes, and seasons.^21,22^

The Baltic Sea is a semienclosed brackish sea, with a large catchment area and a strong terrestrial influence because of river inflow and limited exchange with the North Sea. The Baltic Sea has received a lot of discharge from industry along its coastline, and the ecosystem was severely affected by PCBs in the 1980s.^23^ Today, concentrations of HOCs in surface sediments of the Baltic Sea are decreasing and are far from the peak concentrations observed 40 years ago.^24,25^ There is however a concern that HOCs stored in sediment are released back to water and taken up by fish, which was demonstrated to occur for dioxins in coastal areas of the Baltic Sea.^11^ The Baltic Sea OC cycle is likely to change in the future because of the ongoing actions to reduce loads of nutrients to mitigate eutrophication,^26^ recovery from acidification, and increased transport of terrestrial OC with rivers.^27,28^ It is thus possible that future changes of the OC cycle can influence the HOC flux from air to sediment and thereby reduce the ability of the Baltic Sea sediment to serve as a sink for new or rereleased HOC inputs.

Passive sampling techniques are applied to measure truly dissolved concentrations of HOCs in air, water, and sediment.^29^ The truly dissolved fraction of the chemical is available for diffusive exchange between environmental phases;^30^ thus passive sampling is a well-suited method to study partitioning of HOCs in the environment and the diffusive mass transfer between the environmental matrices like sediment, water, and air.

In this study, we test the hypothesis that OC origin (marine versus terrestrial) has an influence on the HOC fluxes in the Baltic Sea. For this, we perform a case study at two coastal sites in the Gulf of Finland with different relative contributions of terrestrial OC. We used in situ passive sampling to derive freely dissolved concentrations of HOCs in water, sediment (low-density polyethylene passive samplers), and air (XAD passive samplers), to investigate the partitioning, flux rates, and fate of PAHs and PCBs in the sediment–water and air–water interphases at the two sites.

## Materials and Methods

### Chemicals

The chemicals used in this study are presented in Table S1 of the Supporting Information, along with accompanying text in Section 1 Supporting Information.

### Sampling

The study was performed in a long and narrow coastal area (Pohjanpitäjänlahti) of the Gulf of Finland, Baltic Sea. The area receives freshwater from the river Mustionjoki at its far end and brackish water at its opening toward the Baltic Sea (Figure S1, Supporting Information). Deployment of passive samplers and particle traps was performed from 7th of May to 21st of September 2018. Two sampling locations were selected with expected differences in terrestrial influence: one site with an expected higher contribution of terrestrial OC closer to the outlet of the river (terrestrial OC N: 59°55′22.2″, E: 23°20′17.999″, max water depth 13 m) and one site with a higher expected contribution of marine OC closer to the Baltic Sea (marine OC N: 59°51′10.2″, E: 23°15′10.8″, max water depth 25 m) (Figure S1, Supporting Information). The stronger terrestrial influence at the site terrestrial OC compared to the site marine OC is supported by the salinity at different depths (Figure S2, Supporting Information), which was lower at the site with more terrestrial OC (because of the influence of the Mustionjoki river). Settling particles were collected with particle traps having two parallel collection tubes (78 cm^2^) deployed below the photic zone (terrestrial OC 8 m, marine OC 15 m, 134 days) at each site (Figure S3, Supporting Information). Two parallel sediment cores were collected from both sites using a GeMax twin-corer (May 7, 2018), sliced (2 cm), and stored frozen in Nasco Whirl bags at −20 °C until analysis. Water samples for dissolved and total organic carbon (DOC; TOC) analysis from different water depths (terrestrial OC: 2, 5, 7, and 10 m and marine OC: 2, 5, 10, 15, and 20 m) were collected during sampler deployment and retrieval and stored frozen at −20 °C. Subsamples (50 mL) were filtered through a 0.45 μm syringe filter for DOC analysis. Both DOC and TOC were analyzed on a TOC-L Shimadzu total organic carbon analyzer. The average water temperature near the sediment, at 30 meters depth was 6.2 °C (oceanographic monitoring buoy of the Helsinki university research station, Tvärminne Zoological Station N: 59°50′16.8″ E: 23°15′17.4″), and average surface water temperature was 15.9 °C (Finnish Meteorological institute, N: 59°49′12″ E: 23°18′10.2″). Average air temperature was 17.6 °C (N: 59°50′13.2″ E: 23°14′19.2″), and average wind speed was 7.03 m s^–1^ (Finnish Meteorological institute, N: 59°48′11.4″ E: 22°54′16.199″).

#### Sediment–Water Interphase and Water Column Sampling

Low-density polyethylene (LDPE, Ab Rani Plast Oy) was used for water and sediment pore-water sampling (134 days). A sediment penetrating pore-water probe, described in detail by Lin et al.,^31^ deployed at the sea bottom, was used for the sediment–water interphase passive sampling. Briefly, the pore-water probe consists of a stainless-steel frame that supports a hollow stainless-steel tube in the middle. The probe moves freely in vertical direction, and when the frame lands on the sediment bed, the probe continues to penetrate into the sediment. Individual LDPE strips were embedded on the outer surface of the probe in 11 depressions (Figure S3, Supporting Information). The sediment pore-water profile and the overlying bottom water were sampled in 5 cm intervals (approximately 30 cm below and above the sediment surface). Water column sampling was performed by mounting the LDPE samplers on the ropes with twist ties between the bottom weight and the surface buoy (terrestrial OC: 0.5, 5, and 10 m and marine OC: 0.5, 5, and 15 m) (Figure S3, Supporting Information). After deployment, the LDPE passive-sampler strips were collected, biofilm was removed from the strips with a lint-free paper and a few drops of ultrapure water (Milli-Q), and then the strips were stored in 22 mL glass vials (Supelco vials with PTFE liner screw cap) at −20 °C until analysis. Field blanks (*n* = 9) were collected by shortly exposing LDPE strips in the field during deployment, after which the samplers were stored in 22 mL glass vials at −20 °C, and processed together with the other strips.

The interphase between water and sediment was clearly visible on the pore-water probes after deployment. At both sites, the probes had successfully penetrated approximately 30 cm depth into the sediment (Figure S3, Supporting Information). There were some difficulties collecting the pore-water probe at the marine OC site, but this is considered to have a minor effect, as described in detail in Supporting Information Section 2.

#### Air Sampling

Air sampling (99 days, 12th of June to 21st of September 2018) was performed following the method described by Wania et al.^32^ The XAD resin (∼10 g) was packed in stainless-steel mesh cylinders (height 10 cm, diameter 1.5 cm) and deployed in stainless-steel housings described by Wania et al.^32^ in triplicate (Figure S3, Supporting Information). Prior to deployment, the XAD-samplers were precleaned by Soxhlet extraction (1000 mL) with hexane (24 h) and acetone (24 h), dried in a vacuum exicator overnight, and stored at −20 °C until deployment. Samplers were placed on shore, near the water line as close to the aquatic sampling sites as possible, at approximately 1.5 meters height (terrestrial OC: N 59:55:619 and E 23:20:261 and marine OC: N 59:51:004 and E 23:15:279) (Figure S1, Supporting Information). During deployment, three field blanks were collected by shortly exposing XAD-samplers in the field at the terrestrial OC site. Field blanks were stored at −20 °C and processed together with the air samples.

#### Preparation and Analysis of the Passive Sampling Polymer

Prior to sampling, 35 μm-thick LDPE sheets were cut to 2 cm × 30 cm strips and precleaned by soaking in ethyl acetate, methanol, and acetone during 24 h each, after which performance reference compounds (PRCs) were loaded into the strips following a modified method from Birch et al.,^33^ described in detail in Supporting Information Section 3 and Table S2. After deployment, the target analytes were extracted from the samplers, by covering the strips with solvent (heptane/acetone 1:1, v:v) and shaking horizontally (100 rpm). The extraction was repeated twice, each time for a minimum of 12 h. For cleaning, the extract was passed through a silica column (100% activated, 450 °C for 4 h), topped with anhydrous sodium sulfate, and packed in a glass pipette (described further in the Supporting Information). Cleaned sample extracts were reduced in volume under nitrogen flow and analyzed on a GC–MS (Thermo Scientific ISQ LR GC/MS, equipped with a 30 m × 0.25 mm TG-SILMS column of 0.25 μm thickness), using electron impact ionization (EI, 70 eV) in the selected ion monitoring (SIM) mode.

#### Analysis of XAD-Samplers

After deployment, the XAD-samplers (XAD resin inside the metal mesh) were placed in 30 mL extractors, and internal surrogate standards (ISsur) were added, after which the samplers were Soxhlet extracted with acetone (24 h) and hexane (24 h). The solvent fractions were combined, and the extract was reduced in volume to 1 mL under gentle nitrogen flow. The sample cleanup and analysis followed the same procedure as for LDPE samplers. To minimize background contamination, the extractors and collection bottles were cleaned prior to sample extraction by operating the Soxhlet apparatuses empty for 2 × 24 h with acetone and hexane, respectively.

#### Sample Processing of Sediment and Suspended Particle Samples

One sediment core at each location was used for dating, which was performed by analyzing Lead-210 (Pb-210) and complementary analysis of Radium-226 (Ra-226) and Cesium-137 (Cs-137, Flett Research Ltd., Canada). Sample processing and analysis were performed according to previously described methods.^34−36^ A constant rate of supply model was applied to infer the sedimentation rate and the age of the sediment at particular depths. The stable isotope composition of the OC (δ^13^C), TOC, and total organic nitrogen (TON) was determined from the surface layer (0–2 cm) of the second sediment core, and from the settling particles collected with the particle traps (Finnigan DeltaV advantage with CarloErba NC2500 analyzer), following the method from Gustafsson et al.^37^ with small modifications (Section 4 Supporting Information).

A simple end-member-mixing model was used to calculate the contribution of marine and terrestrial OC in sediment at the two sites, assuming that these are the two main sources of carbon (i.e., *f*_ter_ + *f*_mar_ = 1, where *f*_ter_ and *f*_mar_ are the contribution of terrestrial and marine OC in the sediment, respectively):
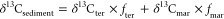
1

Marine organic matter typically has δ^13^C values between −20 and −22‰ and terrestrial vascular plant material between −23 and −30‰.^38,39^ Selected end-member values for δ^13^C_mar_ and δ^13^C_ter_ in the Baltic Sea were −22 and −28‰, respectively, based on the literature.^40,41^

For chemical analysis, surface sediment and settling particles were freeze-dried (Hetosicc, Birkerod Denmark) and grounded to fine powder. Sediment (4 g) and settling particles (2.5 g) were weighed in cellulose extraction thimbles (19 × 90 Munktell and 25 × 90 mm Whatman) and Soxhlet-extracted in 30 mL (sediment) and 50 mL (settling particles) extractors as described above for XAD-samplers. Samples were analyzed in duplicate, and four method blank samples were added for quality control. The cleanup of the sediment and settling particle samples were performed in two steps: (1) for PAHs following the method from Mandalakis et al.^42^ with modifications according to Mustajärvi et al.,^43^ combined with (2) an acid treatment method described in the study by Nybom et al.^44^ to improve PCB recovery. The method for sample processing is described in more detail in the Supporting Information Section 5.

#### Analysis of Lignin Phenols in Sediment

Lignin is a characteristic component of the cell walls of vascular plants;^45^ thus lignin phenols are used as terrigenous biomarkers. An alkaline CuO oxidation protocol described in the study by Goñi and Montgomery (2000)^46^ was applied to extract lignin phenols from surface sediment collected at the two sites (terrestrial OC and marine OC) using a microwave-based method.^47,48^ Approximately 200 mg of dry sediment was loaded in Teflon tubes and mixed with ∼500 mg cupric oxide (CuO) and 50 mg ammonium iron(II) sulfate hexahydrate ((NH_4_)_2_Fe(SO_4_)_2_·6H_2_O) and suspended in N_2_-purged 2 M NaOH solution. The extraction was performed using an UltraWAVE Milestone 215 microwave system at 130 °C for 90 min. An internal volumetric standard (ethyl-vanillin, cinnamic acid) was added after extraction, and concentrated hydrochloric acid (HCl) was added until pH 1. Lignin phenols were extracted using ethyl acetate (EtOAc) through liquid–liquid separation, while residual water was removed using anhydrous sodium sulfate (NaSO_4_). Afterward, samples were dried in a CentriVap (Christ RVC 2-25) at 60 °C for 1 h and then redissolved in pyridine. Aliquots of the total extract were derivatized with bis-trimethylsilyl trifluoroacetamide (BSTFA) + 1% trimethylchlorosilane (TMCS) to silylate exchangeable hydrogen atoms at 60 °C for 30 min. The samples were analyzed using gas chromatography with a mass spectrometer detector (GC–MS 7820A, Agilent Technologies), in the total ion chromatogram mode, using a DB1-MS column (30 m × 0.25 mm; 0.25 μm film thickness). Concentrations of individual lignin phenols and other CuO oxidation products were quantified against five-point concentration curves of external standards. The carbon-normalized lignin content (mg/g OC) refers to the sum of syringyl, vanillyl, and cinnamyl phenols. Determined concentrations of syringyl, vanillyl, and cinnamyl phenols (mg/g OC) are presented in Table S3.

#### Determination of *C*_w_, *C*_pw_, *C*_a,_ and Diffusive Fluxes

Equations to derive concentration and diffusive flux data are described here briefly with more details provided in the Supporting Information Section 6. Freely dissolved water (*C*_w_) and sediment pore-water (*C*_pw_) concentrations were determined using the compound-specific polymer–water partition coefficient (*K*_PE_w_) from Smedes et al.:^49^
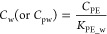
2where *C*_PE_ is the equilibrium passive-sampler concentrations. PRCs were used to assess if equilibrium between the passive sampler and the environment was reached, and if not, to infer exchange rate constants^50,51^ (eqs S2 and S3, Supporting Information). Passive samplers were deployed at different water depths, where also the average temperature during the sampling varied from 6.2 to 15.9 °C. The *K*_PE_w_ was temperature-corrected following the modified van’t Hoff equation (eq S4, Supporting Information),^29^ with the effect being up to 0.16 log units. The effect of water salinity on the HOC solubility and consequently on the polymer–water partition coefficients is considered less significant compared to the effect of temperature,^29,52,53^ and the salinity at the sampling site is low (≤6‰). Therefore, salt correction of the *K*_PE_w_ was not needed.

From the determined sediment pore-water concentrations, diffusive fluxes (*F*) within the sediment were calculated using Fick’s law (eq S4–S9),^54^ and for the fluxes in the sediment–water interphase, the effect of the sediment–water boundary layer was considered (eq S10).^54,55^ A positive net flux indicates a flux direction from *pore-water* to bottom water (*C*_bw_), that is, release of contaminants from sediment to water. If the difference in concentration between phases was less than ×2 (0.30 log units), the system was considered to be at equilibrium, to account for uncertainties from analyses and ambient concentration calculations. The site-specific OC–water partition coefficients (*K*_oc(obs)_) were calculated as a ratio of the carbon-normalized sediment concentrations and the sediment pore-water concentrations in the surface sediment (0–2 cm). Similarly, for the suspended particles, *K*_oc(obs)_ was calculated as a ratio of concentration in the suspended particles collected with particle traps and the water concentration at the deployment depth of the particle traps (eq S11).

The time-averaged air concentrations (*C*_a_) were calculated from the air samplers (*C*_XAD_), according to the study by Wania et al^32^ (eq S12), by applying empirical sampling rates of the XAD resin with 122 days deployment time from Armitage et al.^56^ For analytes for which published data on sampling rates were not available, the sampling rate was estimated from a regression analysis between sampling rates and log *K*_ow_(57,58) (regressions presented in Tables S4 and S5, Supporting Information). The Whitman two-film model was used to calculate the air–water fluxes (eqs S13–S21, Supporting Information).^54^

### QA/QC

Method blanks (solvent only) were included in all sample extraction batches. All samples were blank-corrected with average field blank concentrations when available (sediment–water interphase, water samples, and XAD-air samples) or with method blank average concentrations (sediment and particle trap samples). Limits of detection (LODs) were determined as blank average plus three times standard deviation. Data on concentrations below LODs were excluded from further analysis. The average field blank concentrations for LDPE passive samplers were 1.03 ± 1.36 ng g^–1^ for PAHs and 0.05 ± 0.07 ng g^–1^ for PCBs. The field blank concentrations for XAD-air samplers were 0.20 ± 0.43 and 0.02 ± 0.06 ng g^–1^ and method blank concentrations for sediment and particle traps 6.26 ± 11.06 and 0.13 ± 0.19 ng mL^–1^ for PAHs and PCBs correspondingly. For LDPE passive samplers, 92% (PAH) and 80% (PCB) of the observations were above the LOD. For XAD-air samplers, the observed data above the LOD were the lowest, 63% (PAH) and 66% (PCBs), and for sediment and suspended particle samples, the observed data above the LOD were the highest 95% (PAH) and 89% (PCBs), because of the relatively higher concentrations in sediment compared to passive samplers. Analyte-specific blank concentrations are presented in Tables S6 and S7, Supporting Information. Average ISsur recoveries were 77% for LDPE and 82% for sediment samples and 43 and 35% in particle trap and XAD-air samples, respectively. All analyte concentrations were recovery-corrected. Low ISsur recoveries were observed especially for two–three ring PAHs, which is likely related to losses during vaporization of solvent under N_2_ flow. Analytes with ISsur recoveries ≤20% were excluded from further analysis (Table S8, Supporting Information).

The PRC concentrations (excluding PCB155) in the field blank samples were 89 ± 7% of the nominal spiked concentration indicating a successful spiking of the PRC into the polymer prior sampling. The PRC concentration of PCB155 in the blank samples was only 53% of the nominal spiked concentration, suggesting an error during spiking (see the Supporting Information for details). However, the concentrations in the parallel blank samples were consistent and quantifiable (15–20 times higher than the LOD); thus also PCB155 could be used for determination of the sampling rate (field blank concentrations, *C*^0^_PE_ vs concentration in samplers after deployment, *C*^t^_PE_). The PRCs interfered with the quantification of the native analytes phenanthrene, pyrene, benzo[*a*]pyrene, and PCB8, and because of the analytical issues, these compounds were excluded from the data, and results are presented as ∑PAH_17_ and ∑PCB_21_. The analysis of the PRCs from the deployed samples showed that equilibrium between samplers and water was only achieved in the water column for two- to four-ring PAHs and mono-tetra-chlorinated PCBs (C13-phenanthrene and C13-pyrene, and C13-PCB8, C13-PCB32, and C13-PCB47). The samplers were closer to equilibrium near the water surface. In samplers deployed in the sediment, all PRCs were found after sampling (8–100%), indicating that equilibrium had not been achieved even for the lower molecular-weight compounds. For a reliable determination of the sampling rate, it is recommended that approximately 20–80% of initially loaded PRC concentration should be quantified from the samplers after deployment.^59,60^ Some sampling rates were quantified in this study based on less than 20% or greater than 80% of the respective PRC loss from the samplers (Table S9, Supporting Information).

## Results and Discussion

### Site Characterization

Data of TOC, TON, and δ^13^C in sediment and suspended particles are presented in Table 1. The surface sediment TOC values of 5.68 and 4.65% at the marine OC and terrestrial OC sites, respectively, agree with previously reported TOC from the Gulf of Finland (4.3%^61^). The water TOC content in May and September was 5.29 ± 0.20, 4.62 ± 0.11 mg L^–1^ at the marine OC site and 5.96 ± 0.72, 5.71 ± 0.40 mg L^–1^ at the terrestrial OC site (Figure S4, Supporting Information). Particulate OC constituted ≤10% of the TOC in the water (comparison of DOC and TOC).

**Table 1:** Characteristics of Sediment and Suspended Particles, Total Concentrations of ∑PCB_21_ and ∑PAH_17_ in Surface Sediment and Suspended Particles and Total Lignin Concentration in Surface Sediment at the Two Sites: Marine OC Site and Terrestrial OC Site

	sediment		suspended particles	
	marine OC	terrestrial OC	marine OC	terrestrial OC
TOC (%)	5.68	4.65	10.12	6.73
TON (%)	0.71	0.55	1.45	0.97
C/N (mol/mol)	9.36	9.87	8.12	8.12
δ^13^C (‰)	–22.68	–24.42	–22.24	–24.06
*f*_ter_ (%)	11	40	4	34
sedimentation rate (g cm^–2^ year^–1^)	0.09^a^	0.14^a^	0.08 ± 0.001^b^	0.27 ± 0.06^b^
total Lignin (mg/g_OC_)	3.66	6.97		
∑PCB_21_ (μg kg_OC_^–1^)	65.30 ± 4.74	62.31 ± 4.59	100.27 ± 68.85	81.73 ± 2.95
∑PCB_21_ (μg kg dw^–1^)	3.92 ± 0.41	3.06 ± 0.30	10.82 ± 6.75	6.04 ± 0.13
∑PAH_17_ (mg kg_OC_^–1^)	8.78 ± 0.54	9.21 ± 0.10	2.40 ± 0.05	3.98 ± 0.18
∑PAH_17_ (mg kg dw^–1^)	0.50 ± 0.03	0.43 ± 0.004	0.24 ± 0.01	0.27 ± 0.01

The lower δ^13^C values in both sediment and suspended particles at the terrestrial OC site (−24.06 and −24.42‰) compared to the marine OC site (−22.68 and −22.24‰) support the terrestrial influence to be higher at the terrestrial OC site (Table 1). Accordingly, the end-member model calculated a contribution of terrestrial OC (*f*_ter_) in sediment at the marine OC site to 11% and at the terrestrial OC site to 40% (Table 1). Furthermore, the two sites show contrasting concentrations of carbon-normalized lignin content (mg/g OC), with 6.97 mg g^–1^OC at the terrestrial OC site and 3.66 mg g^–1^OC at the marine OC site, demonstrating higher contribution of terrestrial OC in sediment at the site selected for its higher terrestrial influence.

The vertical sedimentation rates of particulate matter (collected by the particle traps) were a factor of three lower at the marine OC site compared to the terrestrial OC site, and the sedimentation rates determined with dating of sediment organic matter from the top 2 cm are comparable to the measurements from the particle traps (Table 1).

### Sediment, Sediment Pore-Water, Water, and Air Concentrations

Concentrations of PCBs and PAHs in surface sediment, ∑PCB_21_ 3.92 ± 0.41 and 3.06 ± 0.30 μg kg^–1^ dw and ∑PAH_17_ 0.50 ± 0.03 and 0.43 ± 0.004 mg kg^–1^ dw at the marine OC and terrestrial OC sites, respectively (Table 1), are well in line with reported sediment concentrations from the Gulf of Finland; ∑PCB_7_ approximately 3 μg kg^–1^ dw^62^ and ∑PAH_13_ 0.75 mg kg^–1^ dw.^63^ The concentrations of PCBs and PAHs in the surface sediment and settling particles were similar at both sites (within a factor of 1–1.6, Table S10, Supporting Information).

**Figure 1:**
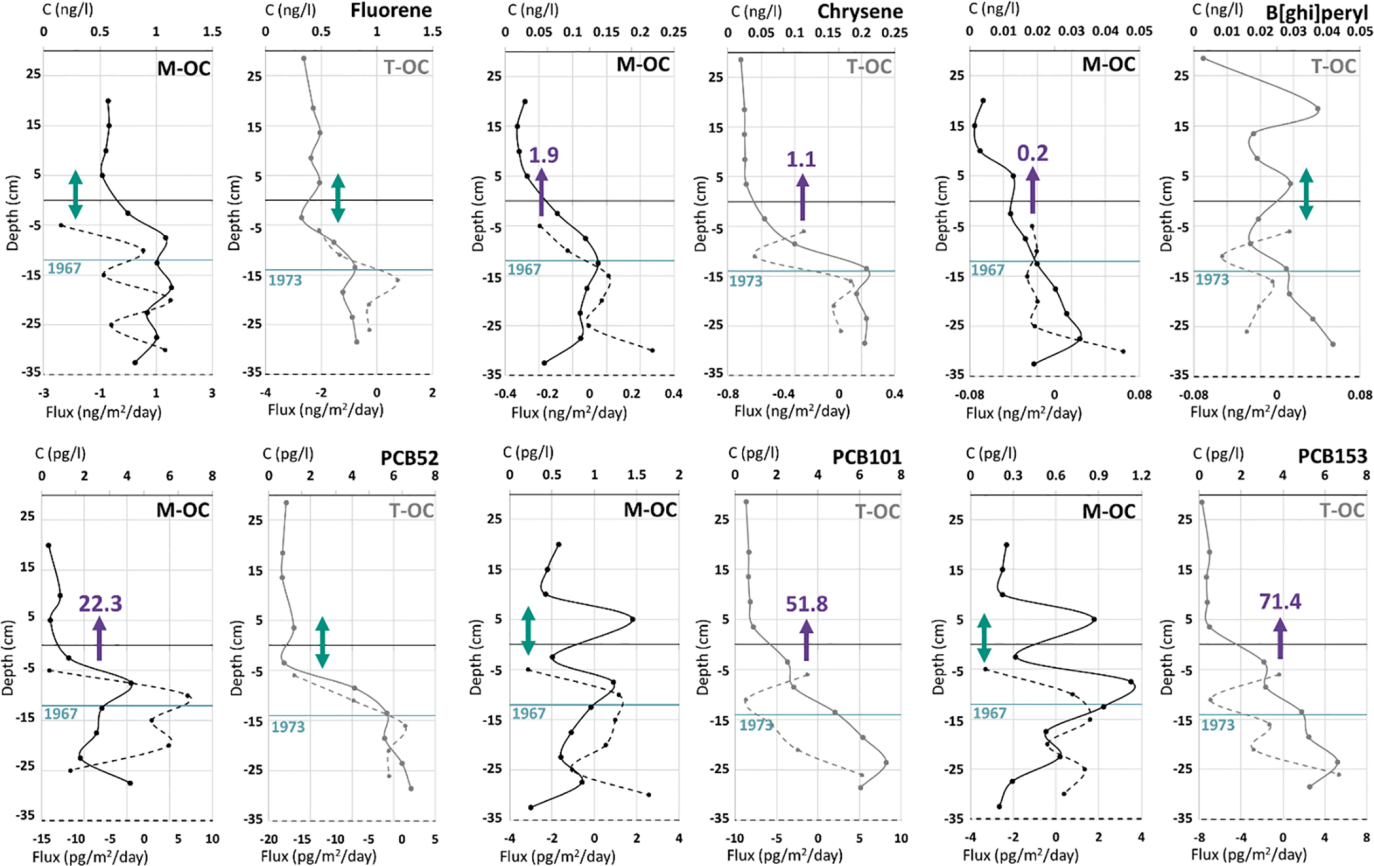
Sediment pore-water concentrations and bottom water concentrations of fluorene, chrysene, and benzo[*ghi*]perylene (ng L^–1^) in the upper panel and PCB52, PCB101, and PCB153 (pg L^–1^) in the lower panel (solid lines) at different depths (cm), at the two sites: marine OC (M-OC) and terrestrial OC (T-OC). Diffusive fluxes (dotted lines) within the sediment between the sequent sediment samples. Negative flux rates indicate upward fluxes from deeper sediment layers toward the surface, and positive flux rates downward fluxes. The sediment–water interphase fluxes are presented with arrows, where the upward arrows indicate fluxes from sediment to water, with the flux rates (ng m^–2^ day^–1^ PAHs and pg m^2–1^ day^–1^ PCBs) indicated next to the arrows. Double-ended arrows indicate that exchange across the sediment–water interface is at or close to thermodynamic equilibrium. Sediment dating at 1967 (M-OC) and 1973 (T-OC) are indicated with a horizontal line.

The freely dissolved concentrations of PAHs in the sediment pore-water were similar at both sampling sites (∑PAH_17_ range from 5.80 to 29.20 ng L^–1^) over the entire sediment profile. The sediment pore-water concentrations of ∑PAH_17_ were the highest at a sediment depth of 13–28 cm (marine OC: 15–25 ng L^–1^) and 14–34 cm (terrestrial OC: ∼15 to 30 ng L^–1^). These sediment layers with the highest HOC concentrations date back to 1960 and before (marine OC) and to the mid-1970s and before (terrestrial OC). The highest pore-water concentrations of PCBs at the marine OC site were found at sediment depths of 8–13 cm (17–22 pg L^–1^), which date back to 1960 to early 1990. At the terrestrial OC site, the pore-water concentrations decreased from the sediment depth 20 cm (37 pg L^–1^) toward the sediment surface, and no clear peak concentration was observed (Figure 1, Figures S4 and S5, Supporting Information). The observed sediment pore-water concentrations are in the same range as those previously reported from the Gulf of Finland: with peak concentrations of ∑PAH_9_ ∼ 19 ng L^–1^^64^ and ∑PCB_7_ ∼ 60 pg L^–1^.^61^

The average concentrations in the water column were 2.51 ± 2.37 and 3.78 ± 4.78 ng L^–1^ ∑PAH_17_ and 38.53 ± 21.68 and 22.02 ± 13.98 pg L^–1^ ∑PCB_21_ at the marine OC and terrestrial OC sites, respectively. For the majority of studied compounds, water concentrations were the highest close to the sediment and decreased toward the water surface (Figures S7 and S8, Supporting Information), suggesting the sediment to be a source of these compounds to water. The observed water concentrations were in the same range as those previously reported for the Baltic Sea: ∑PAH_15_ 1–16 ng L^–1^ for the Baltic Proper near the sediment surface^10^ and ∑PCB_7_ 3–44 pg L^–1^ for the Baltic Proper and the Bothnian Bay in the water column.^65^

The gaseous air concentrations of PCBs were similar at both sites (∑PCB_21_ 17.32 ± 1.13 pg m^–3^ marine OC and 20.76 ± 4.16 pg m^–3^ terrestrial OC). The observed concentrations are lower, but in the same range as those previously reported for the Gulf of Finland: ∑PCB_7_ 20–30 pg m^–3^.^66,67^ For PAHs, only few compounds were quantified (marine OC and terrestrial OC: naphthalene, fluorene, fluoranthene, and chrysene and further terrestrial OC: benzo(*b*)fluoranthene and b(*k*)fluoranthene). The ∑PAH_17_ concentrations were 1.99 ± 1.19 ng m^–3^ at the marine OC site and 1.41 ± 0.88 ng m^–3^ at the terrestrial OC site. Previously reported ∑PAH air concentrations for the Gulf of Finland range from 1 to 10 ng m^–3^.^9,66^ Reported ∑PAH concentrations are influenced by which analytes are included and phenanthrene and pyrene are often among the most abundant PAHs in air.^66^ That these two compounds were not included in this study can explain the lower observed PAH concentrations compared to the literature.^66^

### Fluxes and Site-Specific Sorption of HOCs

At the marine OC site, the ∑PAH_17_ flux rate from sediment to water was 126 ng m^–2^ day^–1^, mainly because of the net fluxes of anthracene, fluoranthene, and benzo(*c*)phenanthrene (Table S10, Supporting Information). At the terrestrial OC site, the sediment pore-water and bottom water were close to equilibrium, with only individual PAHs showing low levels of net sediment-to-water fluxes (Table S10, Supporting Information). At both sites (marine OC and terrestrial OC), the sediment functions as a source of PCBs to water (Table S10, Supporting Information). Flux rates of ∑PCB_21_ were 0.16 ng m^–2^ day^–1^ at the marine OC site and 0.26 ng m^–2^ day^–1^ at the terrestrial OC site. The flux rates observed in this study are one to two orders of magnitude lower than the sediment to water fluxes previously reported for the Baltic Sea, from the mid-1990s to 2015, which were PAHs 300–7000 ng m^–2^ day^–1^^9,10^ and PCBs 3–165 ng m^–2^ day^–1^.^9,13,43^ However, the magnitude of the net flux is highly site-dependent^61,64^ and strongly affected by the sediment HOC concentrations and thus can be expected to vary significantly throughout the Baltic Sea.^10,11,43^

Calculation of air–water fluxes of PAHs was possible for three analytes (fluorene, fluoranthene, and chrysene), and for PCBs, flux rates were calculated for six congeners (PCB4, PCB101, PCB110, PCB118, PCB149, and PCB153) (Table S10, Supporting Information). A negative flux rate (absorption from air to water) was observed for both ∑PAH (−76.54 ng m^2–1^ day^–1^ marine OC and −48.19 ng m^2–1^ day^–1^ terrestrial OC) and ∑PCB (−1.46 pg m^2–1^ day^–1^ marine OC and −2.47 pg m^2–1^ day^–1^ terrestrial OC). Volatilization from water to air was quantified only for the di-chlorinated PCB4 (2.28 pg m^2–1^ day^–1^ marine OC and 2.04 pg m^2–1^ day^–1^ terrestrial OC). Other low-molecular-weight compounds, such as acenaphthene and anthracene, were detected in the surface water but not in air. Previously reported annual diffusive fluxes for the entire Baltic Sea were −1.8 pg m^2–1^ day^–1^ for ∑PCB_7_ and for ∑PAH_15_ −7.4 ng m^2–1^ day^–1^.^9^ Water–air flux rates and directions, being dependent on local emissions and weather conditions, have been shown to represent high spatial and temporal variability and are difficult to compare;^68−70^ however, the observation of the atmosphere being a source, resulting in air-to-water flux dominating for PCBs, is consistent with previous results in the Baltic Sea.^9,65^

#### Site-Specific Observations of HOC Fate

The observed air-to-water fluxes of PCBs were similar at both sites. The PAH flux from air to water at the marine OC site was higher (factor of 1.6), but in the same range as that at the terrestrial OC site. The OC content determined in the settling particles was two times higher at the terrestrial OC compared to the marine OC site (Table 1). Similar air-to-water fluxes at both sites, despite the higher OC content at the terrestrial OC site, indicate that the sorption capacity of the OC (being less at the terrestrial OC site than at the marine OC site) has an effect on the vertical transport and air-to-water flux of HOCs in this study area.

**Figure 2:**
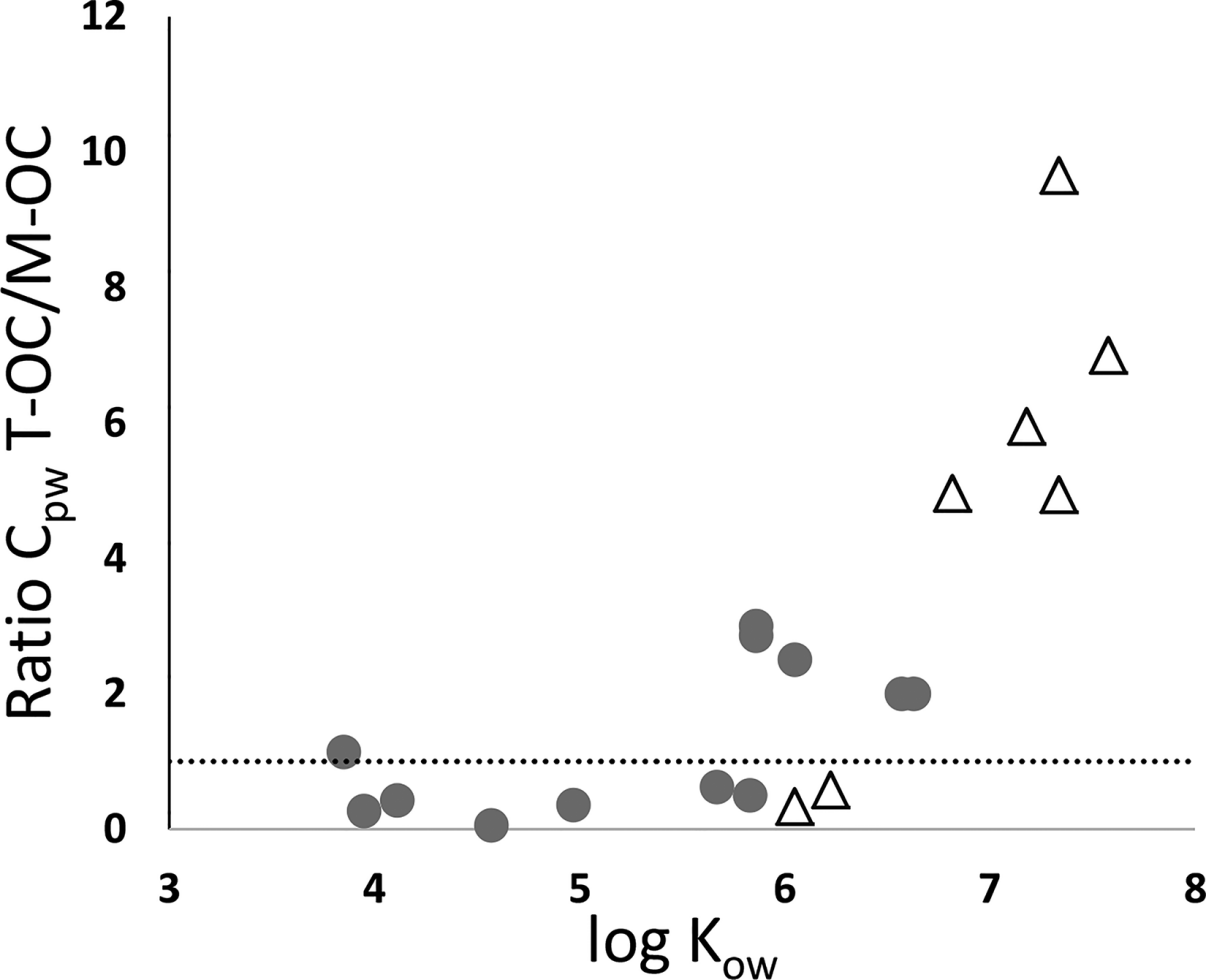
Ratio of pore-water concentrations (*C*_pw_) at the terrestrial OC (T-OC) and marine OC (M-OC) sites of PAHs (gray circles) and PCBs (white triangles) versus the log *K*_ow_ of each compound. The dashed line indicates equal pore-water concentrations at the two sites (*C*_pw_ ratio 1).

Freely dissolved pore-water concentrations of more than four-ring PAHs and penta-heptachlorinated PCBs were higher at the terrestrial OC site compared to the marine OC site (Figure 2). Given that the total concentrations in the surface sediment were similar at the two sites (marine OC *C*_s_OC_/terrestrial OC *C*_s_OC_ range for PAHs 0.7–1.7 and PCBs 0.9–1.7, Table S10, Supporting Information), the lower pore-water concentrations at the marine OC site indicate stronger sorption to the sediment. The difference in pore-water concentrations was more distinct for PCBs compared to PAHs (“marine OC *C*_pw_”/“terrestrial OC *C*_pw_” ratio range for penta-heptachlorinated PCBs between 0.1–0.2 and between 0.3–0.6 for more than four-ring PAHs). Similarly, the sediment pore-water concentrations in deeper sediment layers of the penta-heptachlorinated PCBs were 2–10 times higher at the terrestrial OC compared to the marine OC site (Figure 1, Figure S6, Supporting Information), and one order of magnitude higher sediment-to-water fluxes were observed at the terrestrial OC site. For tri-tetra chlorinated PCBs and less than four-ring PAHs, the pore-water concentrations in the sediment core were in the same range at both sites (“marine OC *C*_pw_”/“terrestrial OC C_pw_” ratios range between 1–2, Figure 1, Figures S5 and S6, Supporting Information). These results suggest that the freely dissolved concentrations in the sediment of penta-heptachlorinated PCBs are constrained by site-specific sorption properties of the sediment, which consequently can limit both sediment-pore-water partitioning and sediment-to-water fluxes.

**Figure 3:**
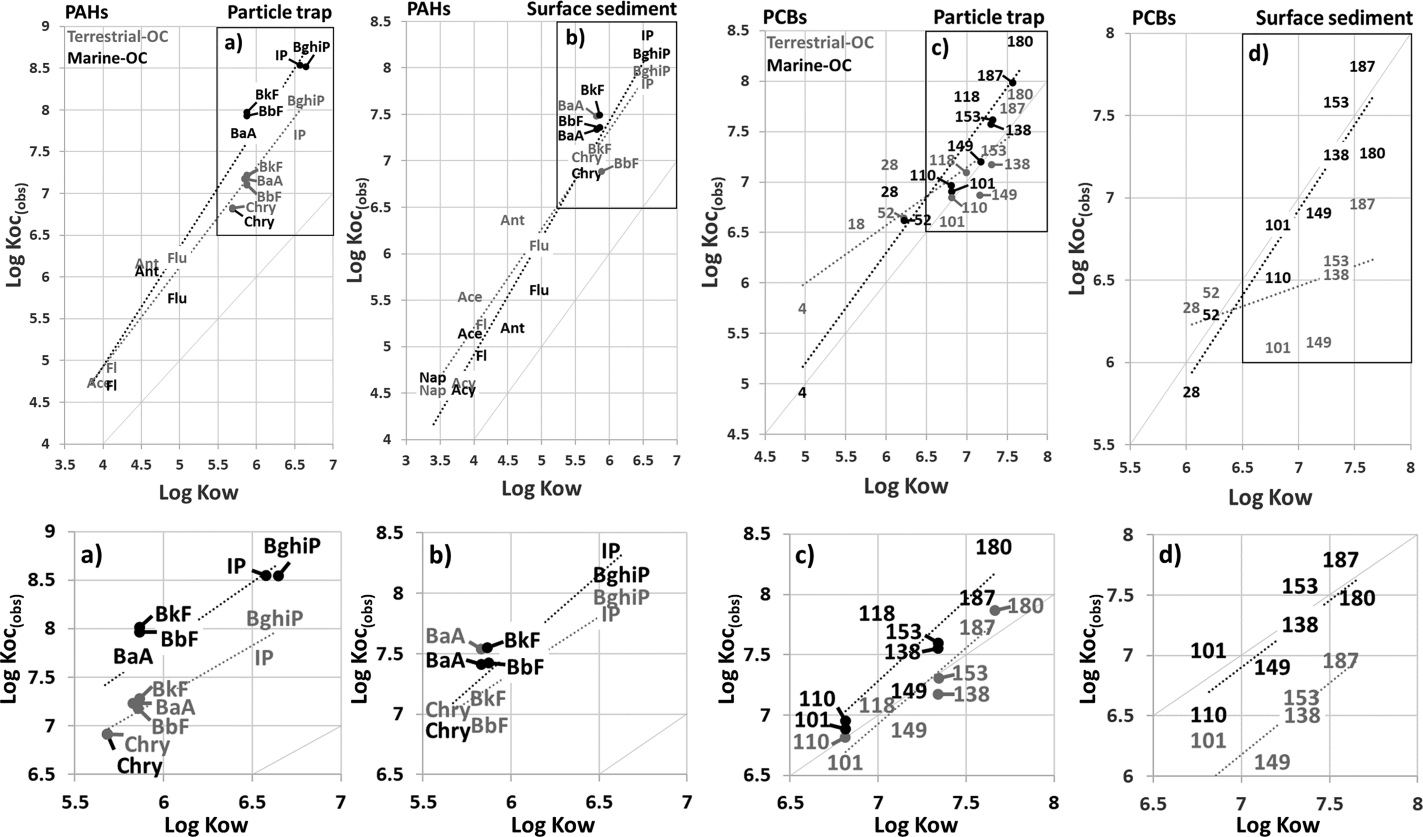
Site-specific partition coefficients for suspended particles and surface sediment for PAHs (left) and PCBs (right). Higher partition coefficients at the marine OC site (black) were observed for PAHs in suspended particles for compounds with log *K*_ow_ > 5.8 (a) compared to the terrestrial OC site (gray), and for PCBs in surface sediment and suspended particles for compounds with log *K*_ow_ ≥ 6.5 (b and c). PCBs are indicated with the congener number, and abbreviations of PAHs are listed in Supporting Information, Table S1. The datapoints are located in the center of the abbreviations, unless indicated with an arrow.

The site-specific partition coefficients between OC and water further demonstrate different sorption capacity of the terrestrial OC and marine OC surface sediment and settling particles. Stronger sorption (i.e., 0.2–1.0 log units higher *K*_oc(obs)_) of more than four-ring PAHs and penta-heptachlorinated PCBs to the sediment and suspended particles was observed at the marine OC compared to the terrestrial OC site (Figure 3, Table S11, Supporting Information). Differences in *K*_oc(obs)_ of penta-heptachlorinated PCBs between the two sites were observed specifically in the surface sediment (0.7–1.0 log unit difference in *K*_oc(obs)_), supporting our hypothesis that OC of marine versus terrestrial origin has different sorption capacity of HOCs. The sorption processes of environmental OC and contaminants are complex.^71,72^ High affinity of natural OC with a high contribution of aliphatic carbon chains (which in marine environments mainly originates from algae biomass) toward HOCs has been observed,^19,20,73^ supporting our results. Lang et al.^61^ observed site-specific sediment–water distribution of PCBs in the Baltic Sea, and although the properties of the OC (such as origin) were not discussed by the authors, the highest sediment–water distribution of PCBs was observed at the Gotland deep which is characterized by high seasonal OC deposition from algae blooms. Similarly, Sobek et al. observed higher sorption of polychlorinated dibenzo-p-dioxins and polychlorinated dibenzofurans (PCDD/Fs) and PCBs to sediment TOC in Baltic Sea offshore compared to coastal sites,^11^ and in a study on PCB concentrations in Baltic Sea sediment, higher concentrations in one of the offshore sites were hypothesized to be caused by higher contribution of marine OC.^24^

### Implications

The complex interactions of cycling of OC and organic contaminants contribute to uncertainties in predictions of the environmental fate of HOCs. The observed differences in sorption affinity of PAHs and PCBs to OC of partly various origin in sediment and suspended particles at the two sites in the Gulf of Finland can help us better understand and reduce such uncertainties. Stronger sorption of more than four-ring PAHs and particularly penta-heptachlorinated PCBs was observed for OC characterized as having a higher contribution of marine origin, in agreement with earlier observations. This leads to reduced sediment pore-water concentrations, and as a consequence, reduced fluxes of those HOCs from sediment to water. Our results suggest that in the water column, not only the total amount of OC but also its origin and thus sorption properties affect the air-to-water flux and vertical transport of HOCs. This conclusion was supported by the observation that air-to-water fluxes were similar at both sites, despite two times higher OC content in water at the site with lower marine influence (terrestrial OC site). According to our results, a future increase of terrestrial OC in the Baltic Sea because of climate change can have consequences for the availability and mobility of high log *K*_ow_ contaminants in sediment, leading to a longer residence time of these hazardous compounds in the circulating marine environment. The effects of climate change on OC cycling however are hard to predict. For instance, climate change can increase seasonal algae blooms, favor specific algae species, reduce the duration of the ice cover, and increase the rainfall, all of which can have individual and combined effects on the fate of HOCs. Further expected dynamics in OC cycling are also expected due to changes in emissions of combustion-derived OC particles from industrial areas, and the subsequent effects on atmospheric-aerosol OC composition, as part of a shift from fossil to biobased fuels and other alternative energy sources.

## Supporting Information Available

Chemicals, description of pore-water probe retrieval, preparation, and analysis of passive sampling polymer including loading of performance reference compounds, stable isotope composition analysis, cleanup method for sediment and suspended particles, determination and calculation of dissolved concentrations in sediment, water and air, fluxes at the air–water interphase, and site-specific partition coefficients (log *K*_oc_) (PDF)
