# Supplementary material for: Effects of Organic Carbon Origin on Hydrophobic Organic Contaminant Fate in the Baltic Sea
Source: Environ Sci Technol. 2021 Sep 23;55(19):13061–71. doi: 10.1021/acs.est.1c04601 (PMC8495899; doi:10.1021/acs.est.1c04601)
Supplement: Supplementary file 1 — es1c04601_si_001.pdf [file es1c04601_si_001.pdf]

Supplementary Information

Effects of organic carbon origin on hydrophobic organic contaminant fate in the Baltic Sea

Nybom, Inna;[1] Horlitz, Gisela;[1] Gilbert, Dorothea;[2] Berrojalbiz, Naiara;[2,3] Martens, Jannik;[1] Arp, Hans Peter H.;[2,4] Sobek, Anna[1*]

[1] Stockholm University, Department of Environmental Science, 10691 Stockholm, Sweden
[2] Norwegian Geological Institute (NGI), P.O. Box. 3930, Ullevål Stadion, N-0806 Oslo, Norway
[3] Department of Environmental Chemistry, IDAEA-CSIC, Jordi Girona 18-26, Barcelona, 08034, Catalunya, Spain
[4] Norwegian university of science and technology (NTNU), Department of Chemistry, NO-7491 Trondheim, Norway

[*]Corresponding author: Anna.Sobek@aces.su.se

Number of pages: 30
Number of additional text pages: 10
Number of Tables: 11
Number of Figures: 8

Content

List of additional text sections

1. Chemicals S2
2. Pore-water probe retrieval from Marine-OC site S8
3. Preparation and analysis of passive sampling polymer S9
3.1 Loading performance reference compounds (PRCs) to passive sampling polymer S9
3.2 Extraction and clean-up of LDPE passive samplers S10
4. Stable isotope composition analysis S11
5. Clean up method for Sediment and Suspended particles S12
5.1 Cleanup of PAH fraction S12
5.2 Cleanup of PCB fraction S12
6. Determination of C_w, C_{pw}, C_a and diffusive fluxes - Calculations S13
6.1 Temperature correction for K_{PE_w} S13
6.2 Diffusive fluxes in the sediment pore-water S14
6.3 Diffusive fluxes in the sediment-water interphase S15
6.4 Site-specific partition coefficients S15
6.5 Fluxes at the air-water interphase S15
References S29

List of Tables

S1 List of external and internal standards, performance reference compounds and solvents S3
S2 Nominal spiked, and determined PRC concentrations in LDPE passive samplers S10
S3 Concentrations of lignin phenols, and total lignin in Terrestrial-OC and Marine-OC sites S13
S4 Physical–chemical properties of analyte PAHs used in the calculations S18
S5 Physical–chemical properties of analyte PCBs used in the calculations S19
S6 Blank concentrations of PAHs in field and method blanks S20
S7 Blank concentrations of PCBs in field and method blanks S21
S8 Recoveries of internal surrogate standards S22
S9 Fraction of PRCs left in the samplers after deployment S22
S10 Measured analyte concentrations and fluxes at different environmental phases S23
S11 Site-specific OC-water partition coefficients S28

List of Figures

S1 Map of the sampling sites and weather observation sites S5
S2 Water salinity at different water depths in Terrestrial-OC and Marine-OC sites S6
S3 Illustrations and figures of the sampling devices S7
S4 Water TOC and DOC profiles in Terrestrial-OC and Marine-OC sites S8
S5 Sediment pore-water-, and bottom water concentrations of selected PAHs S24
S6 Sediment pore-water-, and bottom water concentrations of PCBs S25
S7 Water concentrations of selected PAHs at different water depths S26
S8 Water concentrations of PCBs at different water depths S27

1. Chemicals

Target analytes were PAHs (20) and PCBs (22). An internal surrogate standard (ISsur) mixture containing 10 deuterated PAHs and 7 C13-labeled PCBs was added to the samples prior extraction. Recoveries were determined with internal volumetric standards (ISvol) added to the samples prior analysis, containing two deuterated PAHs and two native PCBs (unfavored substitution position, and therefore rare in the environment). Performance reference compounds (PRCs) were added to the passive samplers prior deployment, including three C13-labeled PAHs, four C13-labeled PCBs and one native PCB. Silica gel 60 (0.063-0.200 mm, Merck Millipore) and anhydrous sodium sulfate (Merck Millipore) were used for the sample cleanup. XAD-2 resin (20-60 mesh, Supelco) was used for air sampling. A detailed list of chemicals and solvents used is provided in Table S1 SI.

Table S1. List of external and internal standards, performance reference compounds and solvents. Abbreviations of native compounds used in figure 3 in brackets.

**External standards -PAHs**		**Internal surrogate standards – PAHs**	
Naphthalene (Nap)	Accustandard	D8 Naphtalene	Larodan solution
Acenaphthylene (Acy)	Accustandard	D8 Acenaphthylene	Sigma-Aldrich
Acenaphthene (Ace)	Accustandard	D10 Fluorene	Cambridge Isotope Laboratories Inc
Fluorene (Fl)	Accustandard	D10 Phenanthrene	Cambridge Isotope Laboratories Inc
Phenanthrene	Accustandard	D10 Anthracene	Cambridge Isotope Laboratories Inc
Anthracene (Ant)	Accustandard	D10 Fluoranthene	Cambridge Isotope Laboratories Inc
Fluoranthene (Flu)	Accustandard	D10 Pyrene	Larodan solution
Pyrene	Accustandard	D12 Benzo[a]anthracene	Larodan solution
Benz[a]anthracene (BaA)	Accustandard	D12 Chrysene	Larodan solution
Chrysene (Chry)	Accustandard	D12 Benzo[a]pyrene	Larodan solution
Benzo[b]fluoranthene (BbF)	Accustandard		
Benzo[k]fluoranthene (BkF)	Accustandard		
Benzo[a]pyrene	Accustandard		
Indeno[123cd]pyrene (IP)	Accustandard		
Diebenz[ah]anthracene	Accustandard		
Benzo[ghi]perylene (BghiP)	Accustandard		
Benzo(c)phenanthrene	Dr. Ehrenstorfer		
Dibenzothiophene	Ultra Scientific		
Benzo(e)pyrene	Dr. Ehrenstorfer		
Perylene	Dr. Ehrenstorfer		
**External standards – PCBs**		**Internal surrogate standards – PCBs**	
PCB 3	Dr. Ehrenstorfer	$C13^{12}$-PCB 28	Greyhound solution
PCB 4 (4)	Dr. Ehrenstorfer	$C13^{12}$-PCB 52	Greyhound solution
PCB 8	Larodan solution	$C13^{12}$-PCB 101	Greyhound solution
PCB 18 (18)	Accustandard	$C13^{12}$-PCB 118	Greyhound solution
PCB 20	Larodan solution	$C13^{12}$-PCB 138	Greyhound solution
PCB 28 (28)	Larodan solution	$C13^{12}$-PCB 153	Greyhound solution
PCB 40	Accustandard	$C13^{12}$-PCB 180	Greyhound solution
PCB52 (52)	Larodan solution		
PCB 53	Larodan solution		
PCB 70	Dr. Ehrenstorfer		
PCB 101 (101)	Larodan solution		
PCB 110 (110)	Dr. Ehrenstorfer		
PCB 118 (118)	Larodan solution		
PCB 128	Ultra Scientific		
PCB 136	Larodan solution		
PCB 138 (138)	Larodan solution		
PCB 149 (149)	Larodan solution		
PCB 153 (153)	Larodan solution		
PCB 170	Larodan solution		
PCB 180 (180)	Larodan solution		
PCB 187 (187)	Ultra Scientific		
PCB 200	Dr. Ehrenstorfer		
**Internal volumetric standards**		**Performance reference compounds (PRCs)**	
D10 Acenapthene	Larodan solution	$C13^{6}$-Phenanthrene	Cambridge Isotope Laboratories Inc
D12 Benzo[k]fluoranthene	Dr. Ehrenstorfer	$C13^{3}$-Pyrene	Cambridge Isotope Laboratories Inc
PCB 30	Dr. Ehrenstorfer	$C13^{4}$- Benzo[a]pyrene	Cambridge Isotope Laboratories Inc
PCB 142	Dr. Ehrenstorfer	$C13^{12}$-PCB 8	Cambridge Isotope Laboratories Inc
		$C13^{12}$-PCB 32	Cambridge Isotope Laboratories Inc
		$C13^{12}$-PCB 47	Cambridge Isotope Laboratories Inc
		$C13^{12}$-PCB 111	Cambridge Isotope Laboratories Inc
		PCB155	Cambridge Isotope Laboratories Inc
**Solvents**			
Acetone (SupraSolv)	Merck Millipore	n-Heptane (SupraSolv)	Merck Millipore
Dichloromethane (Suprasolv)	Merck Millipore	n-Hexane (SupraSolv)	Merck Millipore
Ethyl acetate (SupraSolv)	Merck Millipore	Methanol (Suprasolv)	Merck Millipore

Table S1 continued

**Chemicals used in the analysis of lignin phenols**			
CuO	Sigma-Aldrich	Anhydrous sodium sulphate	Sigma-Aldrich
Ammonium iron (II) sulphate hexahydrate	Sigma-Aldrich	NaOH (≥97.0%)	Sigma-Aldrich
Bis-trimethylsilyl trifluoroacetoamide + 1 % trimethylchlorosilane	Sigma-Aldrich	Ethyl acetate (SupraSolv)	Merck Millipore
Hydrochloric acid, conc.	Merck Millipore	Pyridine (Chromasolv)	Sigma-Aldrich
**External standards – lignin phenols**		**Internal volumetric standards – lignin phenols**	
Vanillin	Sigma-Aldrich	Ethyl-vanillin	Sigma-Aldrich
Acetovanillone	Sigma-Aldrich	Cinnamic acid	Sigma-Aldrich
Vanillic acid	Supelco		
Syringaldehyde	Sigma-Aldrich		
Acetosyringone	Sigma-Aldrich		
Syringic acid	Sigma-Aldrich		
p-Coumaric acid	Supelco		
Ferulic acid	Supelco		
3,5-Dihydroxybenzoic acid	Sigma-Aldrich		

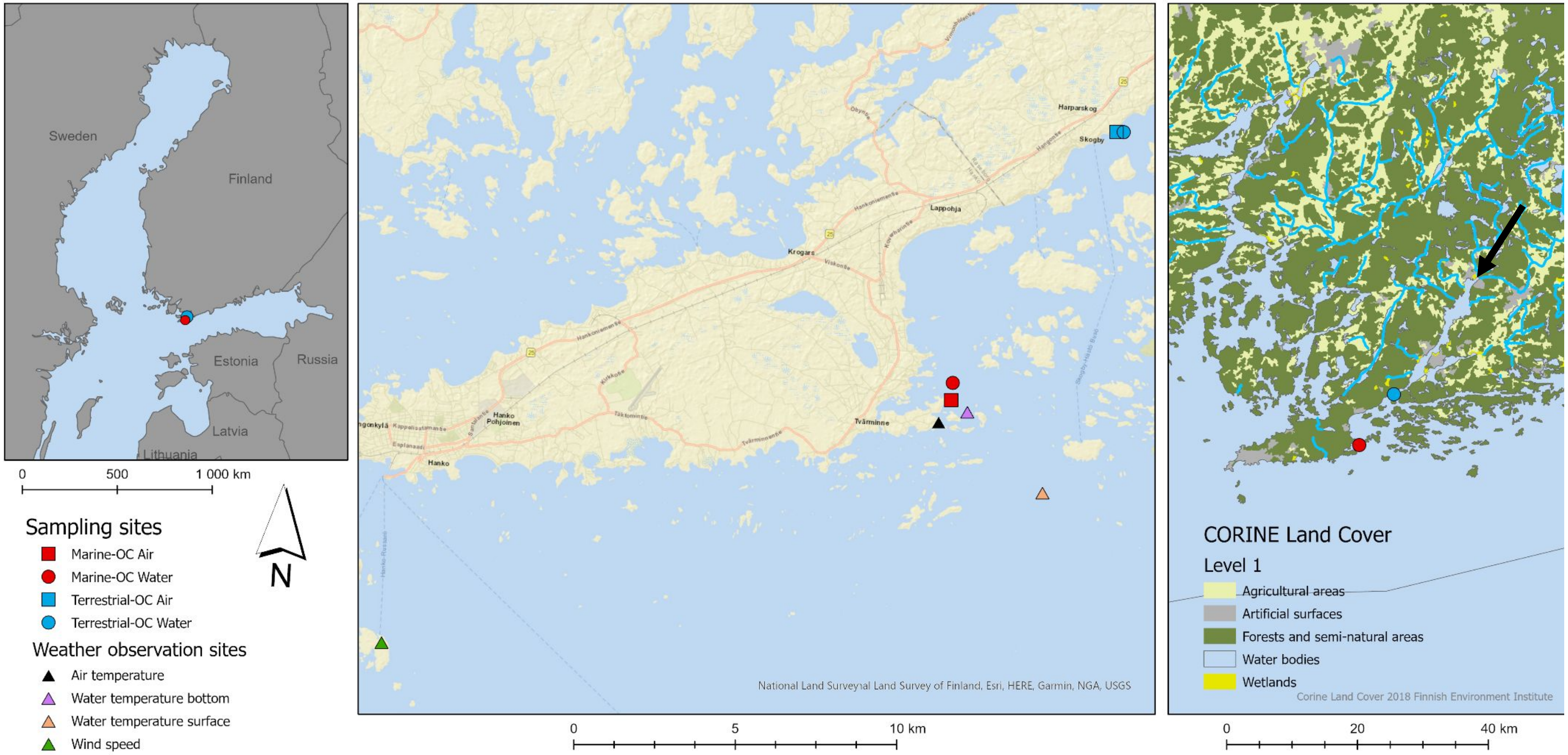

Figure S1. Map of the sampling sites and weather observation sites. The fresh water input via the river Mustionjoki indicated with the black arrow in the map on the right side.

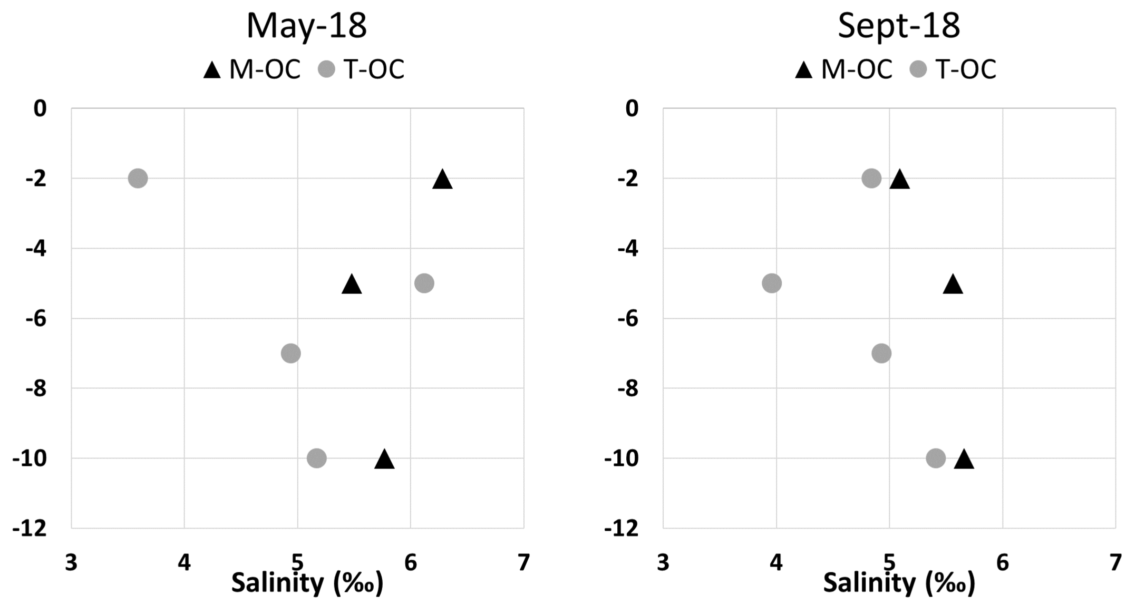

Figure S2. Water salinity at different water depths measured at the beginning (May-18) and at the end of the sampling period (Sept-18) at the two sampling sites Marine-OC (M-OC) black triangles and Terrestrial-OC (T-OC) grey dots. Water salinity at the Marine-OC site was higher compared to the Terrestrial-OC site.

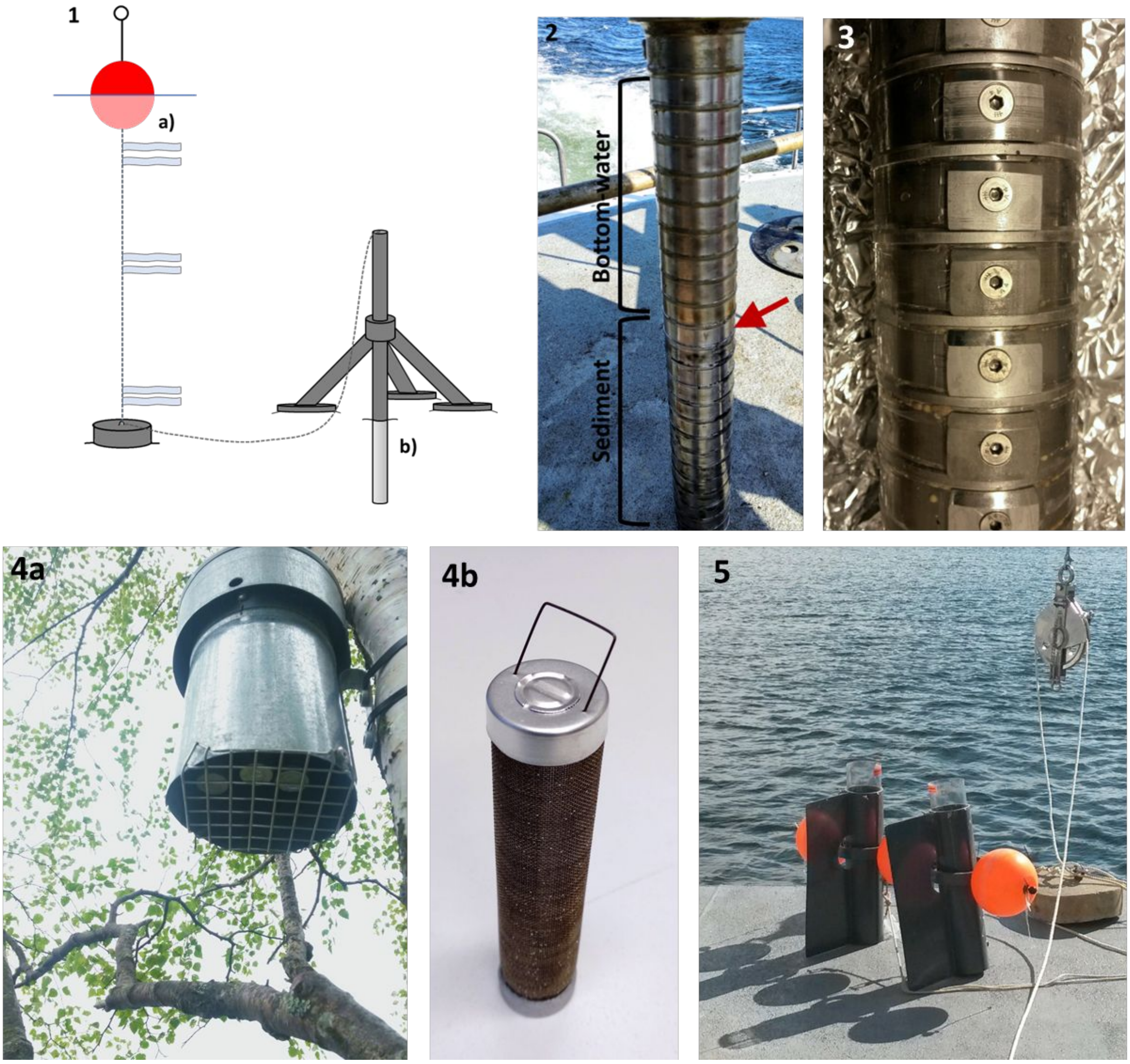

Figure S3. Illustration of the water sampling (a) and bottom-water pore-water probe (b) (1), picture of the probe after deployment (2) and a close up of the passive samplers embedded to the outer surface of the bottom-water pore-water probe on the upper panel (3). Visual difference between samplers exposed to water (top) and sediment (bottom) can be observed (2). The average concentrations from the bottom-water samplers, and the concentrations from the surface sediment sample (red arrow) were used for sediment-water flux calculations. In the lower panel a picture of the air sampling deployment (4a) and the small mesh containing the XAD resin (4b) used for the air sampling. Picture of the particle trap with its two collecting tubes prior deployment on the lower right corner (5).

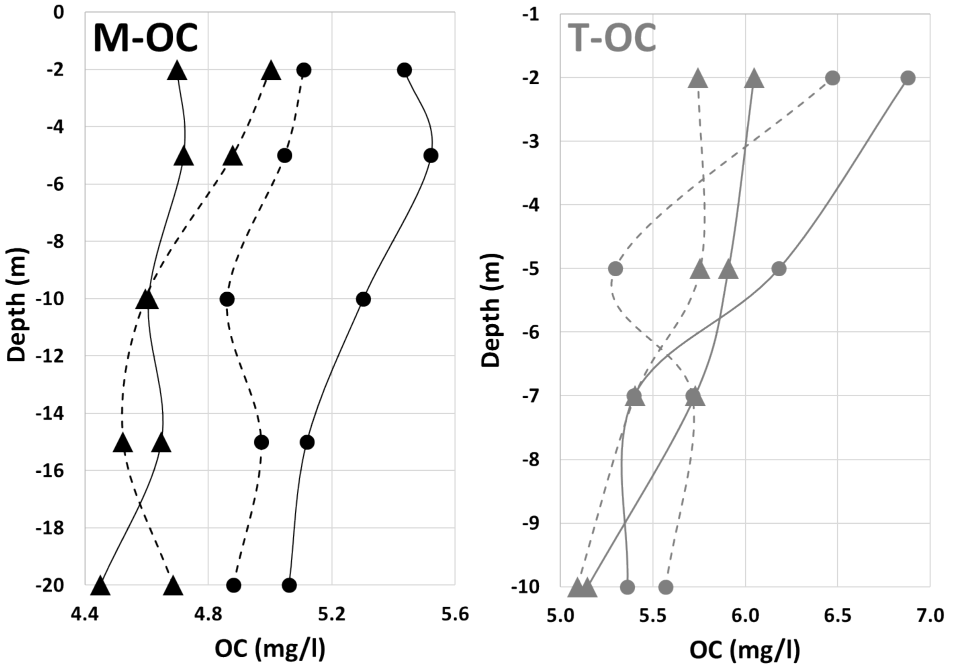

Figure S4. TOC (solid line) and DOC (dashed line) profiles in the water column at the two sampling sites Marine-OC (**M-OC**) and Terrestrial-OC (**T-OC**). Samples were collected at the beginning (May 2018 ●) and at the end of the sampling period (September 2018 ▲). The TOC content decreased from surface to bottom water. Comparison of the non-filtered and filtered samples (0.45 µm) showed that the DOC fraction in the samples was ≥90%. Near bottom and during the second sampling the difference between filtered and non-filtered samples was nominal (i.e. TOC ~ OC).

2. Pore-water probe retrieval from Marine-OC site

A surface buoy was lost during a storm event on the 28th of July 2018. At the end of the passive sampler deployment, the retrieval of the pore-water probe was first tried by dragging an anchor along the sea bottom near the deployment place at two following days. On a third day an under-water ROW camera was used to locate and retrieve the pore-water probe. The camera image revealed that the pore-water probe had tilted on its side during the previous retrieval attempts (likely on the second day; first day the anchor was dragged around the deployment coordinates, on the second day also across the exact deployment location), and the samplers from the sediment pore water were lifted to the sediment surface.

The samplers that were deployed in the sediment have accumulated contaminants during the sampling period and thus have higher chemical concentrations than the bottom water. Hence, when the probe tilted, some of the analytes could start leaking from the samplers when they suddenly were exposed to the bottom water. However, we expect the effect to be minor considering the short exposure time to the bottom water in comparison to the entire sampling

period (maximum 2 out of 134 days). The maximum clearing effect calculated using the sampling rates in the bottom water (PRC clearance rates described below) was 1-3% for PCBs and 1% for PAHs (for the calculation, the probe was assumed to be tilted for 2 days and the maximum clearance rates for the low molecular weight compounds were used). The calculated effect was less than the effect of the average of the blanks.

3. Preparation and analysis of passive sampling polymer

3.1 Loading performance reference compounds (PRCs) to passive sampling polymer

The loading of the PRCs into LDPE passive samplers followed a modified strategy of Birch et al.,[1] where the passive sampling polymer is loaded stepwise forcing the PRCs into the polymer by increasing of water content gradually to >90% in acetone solution.

The total weight of the LDPE polymer strips was noted and strips were placed in an amber glass bottle. The strips were covered with a known amount of acetone (minimum volume of the solvent 5 × the polymer mass, i.e. 50 ml acetone: 10g LDPE, and maximum volume 7% of the total volume of the glass bottle). PRCs were then spiked into the bottle in a pre-prepared stock solution in acetone (end concentration 20 ng g^{-1} PCBs and 340 ng g^{-1} PAHs in LDPE). Polymer strips were left to equilibrate with the acetone solution in a closed bottle, in a shaking table (100 rmp), at room temperature, overnight. Equilibration with solvent swells the polymer slightly, which helps to reach homogeneous spiking of the strips. MilliQ water was then added to the bottle to reach water content of 10%, and after 4 hours the water content was increased to 20%. Polymers were equilibrated in a 20%:80% water:acetone solution overnight. Water content was increased to 33% the following day and after 8 hours increased to 50% v:v and left standing over night. Finally, the water content was increased up to 90% in 4 hour intervals (60% →75% →90%) and left to equilibrate for 24 hours. Prior use the spiking solution was discarded, and the LDPE strips were rinsed two times with clean MilliQ water for 24 hours.

The determined PRC concentrations from blank samples were close to the nominal spiked concentrations (82-114%) for all PRCs excluding the PCB155 (Table S2 SI). Lower PRC concentration of the PCB 155 compared to the nominal concentration (53%) may be due to error in preparation of the spiking stock solution, or indicate that the equilibrium time during spiking was too short. If the water was added too fast, there is a possibility that PRCs did not have enough time to transfer into the samplers, but stick to the spiking bottle walls instead, which would explain the lower attained PRC concentration especially for highly hydrophobic compounds such

as PCB155. However, when the achieved spiked PRC concentration is high enough to be reliably detected, and spiking is homogeneous in the passive samplers the nominal concentration is insignificant, as the sampling rate is determined by a ratio between the determined PRC concentrations in the blank samples and the concentration in the samples after deployment (see equation S2). Standard deviation of the blank sample concentrations (n=3) was small (≤ 6%), confirming homogeneous spiking of the strips. Our results show that the method works for PRC spiking to plastic polymer samplers.

Table S2. Nominal spiked, and determined (± standard deviation) PRC concentrations in LDPE passive samplers (ng/g)

	**C nominal ng g^{-1}**	**C determined ng g^{-1}**	**C_{det}/C_{nom} %**
13C-Phenanthrene	352	335.15 ± 5.81	95
13C-Pyrene	330	270.31 ± 7.03	82
C13-Benzo[a]pyrene	342	309.83 ± 1.76	91
C13-PCB8	20	17.14 ± 0.51	87
C13-PCB32	21	17.45± 1.06	83
C13-PCB47	12	13.22 ± 0.20	113
C13-PCB111	11	12.19 ± 0.28	114
PCB155	20	10.69± 0.47	53

3.2 Extraction and clean-up of LDPE passive samplers

The pre-weighed passive sampler was placed in a 22 ml glass vial (Supelco®, PTFE-liner) and internal surrogate standards were added (Table S1 SI). The passive sampler was covered with an acetone:heptane (1:1, v:v) mixture (volume of the solvent 10 × mass of the polymer, i.e. 5ml solvent mixture for 0.5g LDPE), the vial was capped, and shaken horizontally (100 rpm) in room temperature, in darkness, overnight. The extract was collected with disposable glass pipettes and stored in the freezer (-20 °C). Extraction was repeated for a second time with an equivalent amount of acetone:heptane mixture as in the first phase. The second extract was collected and the extracts were pooled. Finally, the passive sampler was rinsed with the solvent mixture (3 × 0.5 ml) and the rinsing solvent was pooled with the sample extract. Solvent volume was reduced to 1ml under gentle nitrogen flow.

Clean-up columns were built in glass pipettes: a glass pearl (ø 3.8 mm) was placed at the bottom of the pipette and compacted with glass wool. Three cm of silica gel (100% activated, 450°C for 4 hours, stored in n-hexane) was added to the column (approximately 0.5 g) and packed by tapping the column gently from the outside. Silica gel was topped with 0.5 cm anhydrous sodium sulfate (furnished 450°C, 4 hours, stored in n-hexane). The column was

cleaned and conditioned with 5ml of hexane, after which a test tube was placed under the column, and sample was added. When the sample had completely entered the column, 5 ml of n-hexane was added to elute the analytes, followed by 5 ml of hexane:dichloromethane (3:1, v:v), after n-hexane had completely passed in to the column. Care was taken to not let the top of the column dry at any point during the preparation of the column and eluting. The sample was reduced in volume under gentle nitrogen flow and transferred to a GC-vial. Internal volumetric standards (Table S1 SI) were added prior analysis.

4. Stable isotope composition analysis

The $\delta^{13}C$, $\delta^{15}N$, TOC and TON were analyzed with Finnigan DeltaV advantage with CarloErba NC2500 analyzer, in the Stable Isotope Laboratory (SIL) in Stockholm University, Department of Geological Sciences. For the analysis 2 mg of dry material (from the particle trap) or 10 mg (sediment) was weighed to a silver capsule. The samples were acidified in order to remove carbonates. To wet the samples, 25 µl water were added to the silver capsules followed by 25 µl 1 M HCl. The samples were kept at room temperature for 1 hour and were dried in an oven at 40°C. The samples were then treated again with 50 µl of 1 M HCl, left in room temperature for 1 hour and dried at 40°C. The treatment was repeated until effervescence upon acid addition ceased.

Isotopic data is expressed in conventional delta notation (‰):

$$\delta^{13}C_{sample} \text{ or } \delta^{15}N_{sample} = \left(\frac{R_{sample}}{R_{reference}} - 1\right) \times 1000 \quad \text{eq. S1}$$

were R is $^{13}C/^{12}C$ or $^{15}N/^{14}N$ ratio, and the reference is carbonate from the PeeDee Belimnite formation (PDB) for $\delta^{13}C$ and atmospheric N_2 for $\delta^{15}N$.

5. Clean up method for Sediment and Suspended particles

The cleanup of the sediment and suspended particle samples was done in two steps: 1) for PAHs following the method from Mandalakis et al.[2] with modifications according to Mustajärvi et al.,[3] and 2) combined with acid treatment method described in Nybom et al.[4] for PCBs.

5.1 Cleanup of PAH fraction

After extraction, the samples were reduced in volume to 1ml, after which 3ml of pentane was added and the sample was extracted with 2 × 3ml of dimethylformamide (DMF, 5% MilliQ v/v). In order to improve the PCB recovery, the pentane phase was saved for further processing (described below). The DMF phase was extracted by adding 5ml of water and 3ml of hexane, hexane was collected and the extraction was repeated with 2 × 2ml of hexane. The sample was reduced in volume to 1ml and passed through a 2.5 cm silica column (1 cm Ø, 10% MilliQ w/w) topped with Na_2SO_4 (0.5 cm). The column was cleaned and conditioned with 20 ml hexane, and the samples were eluted with 17 ml of hexane. The samples where evaporated to final volume under gentle nitrogen flow, a volumetric standard (IS_{vol}) was added to half of the samples, and the samples were analyzed with GC-MS as described for the LDPE samples.

5.2 Cleanup of PCB fraction

For acid cleanup, the solvent was changed from pentane to hexane, by adding 2ml of hexane and evaporating to 1ml. The samples were shaken with 2ml of MilliQ water, the hexane fraction was collected and passed through Na_2SO_4. The water fraction was further extracted with 2 × 2ml hexane and the extracts were pooled. Samples were evaporated to 2 ml and 1 ml of concentrated sulfuric acid (98% H_2SO_4, EMSURE, Supelco) was added. The samples were shaken, hexane was collected and the acid fraction was further extracted with 2 × 2 ml hexane. Extracts were pooled, and 1ml H_2SO_4 was added once more for further cleanup. The acid fraction was removed carefully, and samples were reduced in volume to 1 ml under nitrogen flow. Finally, the samples were passed through a 2 cm Na_2SO_4 microcolumn in a glass pipette. The column was first cleaned and conditioned with 2 ml of hexane and eluted with 3 × 2 ml hexane. The samples were evaporated and combined with the PAH-fraction extracted previously. IS_{vol} was added to the second half of duplicate samples and all samples were analyzed for PCBs with GC-MS as described for the LDPE samples.

Table S3. Concentrations of lignin phenols Syringyl (S), Vanillyl (V) and Cinnamyl (C), and total lignin as the sum of above mentioned (mg gOC) in the surface sediment from two sites, Terrestrial-OC and Marine-OC.

	Lignin phenol concentrations			
	S	V	C	Total Lignin
	mg gOC	*mg gOC*	*mg gOC*	*mg gOC*
Terrestrial-OC	2.01	2.51	2.45	6.97
Marine-OC	0.65	1.21	1.8	3.66

6. Determination of C_w, C_{pw}, C_a and diffusive fluxes - Calculations

PRC concentrations were used to infer exchange rate constants for passive samplers that had not reached equilibrium between water or sediment pore-water, and the LDPE passive sampler. The elimination rate constant (k_e) was calculated from the clearance rate of PRCs (hr^{-1}):[5]

$$k_e = ln\left(\frac{C^0_{PE}}{C^t_{PE}}\right) \times t^{-1} \quad \text{eq. S2}$$

where t is the sampling time (h), C^0_{PE} is the concentration of PRC in the sampler before deployment (average field blank) and C^t_{PE} is the concentration of the PRC in the sampler at time t. Assuming equal exchange rate of elimination and uptake, the water concentrations of non-equilibrated analytes can be calculated as:[5,6]

$$C_W = \frac{C_{PE}}{\left(1 - e^{-k_e t}\right) K_{PE\_w}} \quad \text{eq. S3}$$

6.1 Temperature correction for K_{PE_w}

Temperature corrections of K_{PE_w} followed the modified van't Hoff equation:[7]

$$K_{PE\_w}(T) = K_{PE\_w}(298) e^{(\Delta H_{PE\_w}/R)\left\{\frac{1}{298} - \frac{1}{T}\right\}} \quad \text{eq. S4}$$

where K_{PE_w} (T) and K_{PE_w} (298) are PE-water partition coefficients at temperature T (K) and at 298 K. R is the universal gas constant (8.3143 J/mol/K), and ΔH_{PE_w} is the enthalpy of PE-water partitioning (kJ/mol). Experimental data for ΔH_{PE_w} is limited, therefore a default value of ΔH_{PE_w} of −25 kJ/mol was used as suggested by Lohmann.[7]

6.2 Diffusive fluxes in the sediment pore-water

Diffusive fluxes (F) within the sediment were calculated using Fick´s law:[8]

$$F = -\Phi D_{wz} \frac{(C_{w1} - C_{w2})}{(z_2 - z_1)} \text{ eq. S5}$$

where Φ is porosity, calculated as a fraction of the volume of water in the sample and the total volume of the sample. D_{wz} is the diffusivity of the compound of interest in the sediment core at depth z, and C_{w1} and C_{w2} are the concentrations in the aqueous phase at depths z_1 and z_2. The diffusivity of the compound in the sediment was calculated as a ratio of the temperature corrected molecular diffusion coefficient (D_{wt}) and the square of tortuosity.[8,9]

The diffusivity of a compound in the sediment (D_{wz}), was calculated as a ratio of the molecular diffusion coefficient D_{wt} and the square of tortuosity (θ):[8,9]

$$D_{wz} = \frac{D_{wt}}{\theta^2} \text{eq. S6}$$

Tortuosity, which is the average distance a molecule travels in a porous media in relation to direct distance, in the direction of the diffusion, can be further expressed in relation to the porosity (Φ):[9]

$$\theta^2 = 1 - 2 \times ln(\Phi) \text{eq. S7}$$

Molecular diffusion coefficients D_w at 25 °C were calculated from target compound molecular weights (M), with:[8]

$$D_w = \frac{2.7 \times 10^{-4}}{M^{0.71}} \text{ eq. S8}$$

and further corrected for the average temperature at the bottom during sampling (6°C): [8]

$$D_{wt} = D_w(1 - 0.01 \times (TD_{25-20°C} \times 5 + TD_{20-15°C} \times 5 + TD_{15-10°C} \times 5 + TD_{10-5°C} \times \Delta T)) \text{eq. S9}$$

where ΔT is the average temperature during sampling subtracted from the temperature 10°C (i.e. 10°C - 6°C = 4), and temperature adjustment coefficients $TD_{25\text{-}20°C}$ 2.45, $TD_{20\text{-}15°C}$ 2.54, $TD_{15\text{-}10°C}$ 2.63, $TD_{10\text{-}5°C}$ 2.72.

D_w at 25 °C, at surface water temperature 16 °C and bottom water temperature 6 °C are presented in Tables 2-3SI.

6.3 Diffusive fluxes in the sediment-water interphase

The flux at the sediment-water interphase is controlled by the thickness of the boundary layer (b), and was determined as:[8,10]

$$F = D_{wt}\left(\frac{C_{pw} - C_{bw}}{b}\right) \text{ eq. S10}$$

where C_{pw} is the concentration in sediment pore-water and C_{bw} is the concentration in bottom water. C_{bw} was determined as an average of 4 (Terrestrial-OC) or 5 (Marine-OC) concentrations from samples deployed at 1-30 cm above the sediment surface. The sampler deployed near the sediment surface was used to determine C_{pw} (depth Marine-OC site 0.5 - 2.5 cm, Terrestrial-OC site 1-3.5 cm) (Fig S2, SI). The boundary layer thickness of 0.0007 m was used in the calculations according to Eek et al.[11]

6.4 Site-specific partition coefficients

For comparison of the two sites, site specific OC-water partition coefficients ($K_{oc(obs)}$) were calculated:

$$K_{oc(obs)} = \frac{C_{S\_oc\ (or\ PT\_oc)}}{C_{pw\ (or\ w)}} \text{eq. S11}$$

where C_{S_oc} is the OC normalized analyte concentration in the surface sediment (0-2 cm) or in the suspended particles (C_{PT_oc}), C_{pw} is the freely dissolved pore-water concentration near the sediment surface and C_w the water concentration at the deployment depth of the particle traps.

6.5 Fluxes at the air-water interphase

The time-averaged air concentrations (C_a) were calculated from the extracted concentrations in the XAD-resins (C_{XAD}) with:[12]

$$C_a = \frac{C_{XAD}}{R_{sr}t} \text{eq. S12}$$

where R_{sr} is the sampling rate of the system ($m^3 day^{-1}$) and t is the sampling time (days). Empirical sampling rates with 122 days deployment time from Armitage et al.[13] were used for the calculation. For analytes for which published data on sampling rates were not available, the sampling rate was estimated from a LFER between sampling rate and log K_{ow}[14,15] (regressions presented in Tables S2-S3 SI).

The air-water fluxes were calculated with the Whitman two-film model:[8]

$$F = K_{ol}\left(C_w - \frac{C_a}{H'_T}\right) \text{eq. S13}$$

where K_{ol} is the total mass-transfer rate coefficient (m day^{-1}), C_w is the dissolved water concentration near the water surface, and C_a is the gas phase air concentration. H'_T is temperature corrected compound-specific Henry's law constant. The van't Hoff equation described in SI (eq. S4 SI) was used to adjust the Henry's law constants to the average surface water temperature during sampling (16 °C) (Tables 2-3 SI). Henry´s law constants at 25 °C, as well as the enthalpies of vaporization were attained from literature.[16,17]

The mass transfer rate coefficient K_{ol} in eq. S13 can be expressed in relation to the air-side mass transfer coefficient (K_a) and water side mass transfer (K_w):

$$\frac{1}{K_{ol}} = \frac{1}{K_a H'_T} + \frac{1}{K_w} \text{eq. S14}$$

The mass transfer coefficients in air and water were calculated as described in Hornbuckle et al:[18]

$$K_a = K_{a(H2O)}\left(\frac{D_a}{D_{a(H2O)}}\right)^{0.67} \text{eq. S15}$$

where D_a and the $D_{a(H2O)}$ are the diffusivities of target analytes and water in air respectively, and the air-side mass transfer coefficient of water (K_{aH2O}) is calculated according to Schwarzenbach et al.,[8] using the average wind speed at 10 meters (u_{10}). Data on wind speed during the sampling period was from the Finnish Meteorological institute (7.03 m/s).

$$K_{a(H2O)} = 0.2 \times u_{10} + 0.3 \text{eq. S16}$$

The D_a and $D_{a(H2O)}$ at 25°C (298K) were calculated from the empirical correlation with the molecular mass (M) and corrected for air temperature during sampling (17.6°C) with $T^{1.724}$ according to Schwarzenbach et al.[8]

$$D_a = \frac{1.55}{M^{0.65}} \text{eq. S17}$$

$$D_{a(298)} = D_{a(T)} \times \left(\frac{298K}{T}\right)^{1.724} \text{ eq. S18}$$

D_a at 25°C and at air temperature 17.6 °C for PAHs and PCBs are presented in Tables S3-S4 SI.

The water-side coefficient in eq. S16 SI was calculated using K_w for CO_2 ($K_{w(CO2)}$) and Schmidt number for target analytes (Sc) and for CO2 ($Sc_{(CO2)}$).

$$K_w = K_{w(CO2)} \left(\frac{Sc}{Sc_{(CO2)}}\right)^{-1/2} \text{eq. S19}$$

The $K_{w(CO2)}$ is related to the wind speed at 10 meters (u_{10}) with:

$$K_{w(CO2)} = 0.45 \times {u_{10}}^{1.64} \text{eq. S20}$$

and the Sc were derived from the relationship of the water viscosity (v_w) at 16°C (1.11×10^{-2} cm s^{-1}) and temperature corrected compound specific diffusivities in water (D_{wt}, from eq. S9 SI).

$$Sc = \frac{v_w}{D_{wt}} \text{eq. S21}$$

Table S4. Log K_{ow}, XAD sampling rates, water and air diffusivities and Henry´s law constants at 25°C and sampling temperatures for PAHs.

	Log K_{ow}[a]	XAD sampling rate $m^3\ d^{-1}$	D_w cm s^{-1} 25°C	D_w cm s^{-1} 16°C (surface)	D_w cm s^{-1} 6°C (bottom)	D_a cm s^{-1} 25°C	D_a cm s^{-1} 17.6°C (air)	H´Pa $mol^{-1}\ m^{-3}$ 25°C[c]	ΔH´[c]	H´Pa $mol^{-1}\ m^{-3}$ 16°C
Naphtalene	3.40	1.53[b]	8.61E-06	6.68E-06	4.43E-06	6.61E-02	6.33E-02			
Acenapthene	3.95	1.49[b]	7.55E-06	5.86E-06	3.88E-06	5.86E-02	5.61E-02	18.5	51.9	68.2
Acenapthylene	3.85	1.19	7.62E-06	5.91E-06	3.92E-06	5.91E-02	5.66E-02	12.7	52.5	24.7
Fluorene	4.11	0.93[b]	7.16E-06	5.55E-06	3.68E-06	5.58E-02	5.35E-02	9.81	48.4	18.1
Dibenzothiophene			6.65E-06	5.16E-06	3.42E-06	5.22E-02	5.00E-02			
Phenanthrene	4.47	0.81[b]	6.81E-06	5.28E-06	3.50E-06	5.34E-02	5.11E-02	4.29	47.3	7.8
Anthracene	4.57	1.04	6.81E-06	5.28E-06	3.50E-06	5.34E-02	5.11E-02	5.64	46.8	10.2
Fluoranthene	4.97	0.69[b]	6.23E-06	4.83E-06	3.20E-06	4.91E-02	4.71E-02	1.96	38.7	3.2
Pyrene	5.01	0.86[b]	6.23E-06	4.83E-06	3.20E-06	4.91E-02	4.71E-02	1.71	42.9	2.9
Benzo[c]phenanthrene			5.71E-06	4.43E-06	2.94E-06	4.54E-02	4.35E-02			
Benzo[a]anthracene	5.83	0.77	5.71E-06	4.43E-06	2.94E-06	4.54E-02	4.35E-02	1.22	66.4	2.8
Chrysene	5.67	0.93[b]	5.71E-06	4.43E-06	2.94E-06	4.54E-02	4.35E-02	0.53	100.9	1.9
Benzo[b]fluoranthene	5.86	0.77	5.32E-06	4.13E-06	2.74E-06	4.26E-02	4.08E-02			
Benzo[k]fluoranthene	5.86	0.77	5.32E-06	4.13E-06	2.74E-06	4.26E-02	4.08E-02			
Benzo[e]pyrene			5.32E-06	4.13E-06	2.74E-06	4.26E-02	4.08E-02			
Benzo[a]pyrene	6.05	0.72	5.32E-06	4.13E-06	2.74E-06	4.26E-02	4.08E-02			
Perylene			5.32E-06	4.13E-06	2.74E-06	4.26E-02	4.08E-02			
Indeno[123cd]pyrene	6.57	0.61	4.99E-06	3.87E-06	2.57E-06	4.01E-02	3.84E-02			
Dibenz[a,h]anthracene			4.96E-06	3.85E-06	2.55E-06	3.99E-02	3.82E-02			
Benzo[ghi]perylene	6.63	0.60	4.99E-06	3.87E-06	2.57E-06	4.01E-02	3.84E-02			

[a]Ma et al. 2010,[15] [b]Armitage et al. 2013,[13] [c]Bamford et al. 1999[17]

Table S5. Log K_{ow}, XAD sampling rates, water and air diffusivities and Henry´s law constants at 25°C and sampling temperatures for PCBs.

	Log K_{ow}[a]	XAD sampling rate $m^3\ d^{-1}$	D_w cm s^{-1} 25°C	16°C (surface)	6°C (bottom)	D_a cm s^{-1} 25°C	17.6°C (air)	H´Pa $mol^{-1}\ m^{-3}$ 25°C[c]	∆H´[c]	H´Pa $mol^{-1}\ m^{-3}$ 16°C
PCB3	4.855	1.01	6.54E-06	5.08E-06	3.37E-06	5.14E-02	4.92E-02	18.9	56	38.5
PCB4	4.965	0.98	5.81E-06	4.51E-06	2.99E-06	4.61E-02	4.41E-02	23	50	43.4
PCB8	5.308	0.90	5.81E-06	4.51E-06	2.99E-06	4.61E-02	4.41E-02	25.7	44	45.0
PCB18	5.633	0.59[b]	5.24E-06	4.07E-06	2.70E-06	4.20E-02	4.02E-02	25.3	35	39.5
PCB20	5.795	0.79	5.24E-06	4.07E-06	2.70E-06	4.20E-02	4.02E-02	29	41	48.8
PCB28	6.048	0.78[b]	5.24E-06	4.07E-06	2.70E-06	4.20E-02	4.02E-02	36.5	33	55.5
PCB40			4.80E-06	3.72E-06	2.47E-06	3.87E-02	3.71E-02	29.7	30	43.5
PCB52	6.223	0.93[b]	4.80E-06	3.72E-06	2.47E-06	3.87E-02	3.71E-02	31.3	31	46.4
PCB53	7.335		4.80E-06	3.72E-06	2.47E-06	3.87E-02	3.71E-02	44.8	25	61.6
PCB70	6.43	0.65	4.43E-06	3.44E-06	2.28E-06	3.60E-02	3.45E-02	32.3	29	46.7
PCB101	6.815	0.52[b]	4.43E-06	3.44E-06	2.28E-06	3.60E-02	3.45E-02	43.2	30	63.2
PCB110	6.815	0.56	4.43E-06	3.44E-06	2.28E-06	3.60E-02	3.45E-02	42	38	68.1
PCB118	6.995	0.52	4.43E-06	3.44E-06	2.28E-06	3.60E-02	3.45E-02	36.3	50	68.5
PCB128			4.13E-06	3.20E-06	2.12E-06	3.37E-02	3.23E-02	32.7	118	146.5
PCB136			4.13E-06	3.20E-06	2.12E-06	3.37E-02	3.23E-02	81.5	27	114.9
PCB138	7.335	0.45	4.13E-06	3.20E-06	2.12E-06	3.37E-02	3.23E-02	45.2	87	136.5
PCB149	7.177	0.48	4.13E-06	3.20E-06	2.12E-06	3.37E-02	3.23E-02	68.4	46	122.7
PCB153	7.335	0.45	4.13E-06	3.20E-06	2.12E-06	3.37E-02	3.23E-02	54	66	124.9
PCB170	7.659	0.39	3.87E-06	3.00E-06	1.99E-06	3.18E-02	3.04E-02	19.4	164	155.9
PCB187	7.566	0.37	3.87E-06	3.00E-06	1.99E-06	3.18E-02	3.04E-02	65.9	96	223.2
PCB180	7.659	0.37	3.87E-06	3.00E-06	1.99E-06	3.18E-02	3.04E-02	37.3	144	232.5
PCB200	7.838	0.33	3.65E-06	2.83E-06	1.88E-06	3.01E-02	2.88E-02	97.5	145	615.5

[a]Sabljic et al. 1993,[14] [b]Armitage et al. 2013,[13] [c]Bamford et al. 2002[16]

Table S6. Blank concentrations (average ± standard deviation) of PAHs in field and method blanks. Field blank concentrations in LDPE passive samplers and XAD -air samplers are expressed as both ng/g and ng/ml for comparability with method blanks.

	**LDPE – passive sampling polymer**			**XAD - air**			**Sediment and particle trap**
	Field blank		method blank	Field blank		method blank	method blank
	ng/g	ng/ml	ng/ml	ng/g	ng/ml	ng/ml	ng/ml
Naphtalene	4.83 ± 1.34	3.59 ± 2.11	2.58 ± 1.61	1.83 ± 0.69	91.68 ± 34.59	37.14 ± 18.89	51.89 ± 26.92
Acenapthene	0.38 ± 0.10	0.26 ± 0.06	0.17 ± 0.07	nd	nd	0.98 ± 0.52	1.56 ± 0.46
Acenapthylene	1.36 ± 0.55	0.99 ± 0.74	0.83 ± 0.75	0.20 ± 0.21	10.23 ± 10.31	4.18 ± 1.52	1.79 ± 1.10
Fluorene	1.73 ± 0.55	1.10 ± 0.15	0.53 ± 0.12	0.04 ± 0.01	1.76 ± 0.26	1.84 ± 0.46	2.43 ± 0.90
Dibenzothiophene	nd	Nd	0.83 ± 0.75	0.08 ± 0.06	4.04 ± 2.76	0.66 ± 0.60	1.05 ± 0.93
Anthracene	0.08 ± 0.02	0.21 ± 0.20	nd	0.06 ± 0.006	3.13 ± 0.24	2.94 ± 0.16	3.05 ± 0.42
Fluoranthene	0.17 ± 0.09	0.17 ± 0.1	nd	0.07 ±0.01	3.56 ±0.53	3.61 ± 0.43	5.81 ± 2.88
Benzo[c]phenanthrene	0.33 ± 0.30	0.23 ± 0.14	0.10 ± 0.16	0.16 ± 0.07	8.06 ± 3.52	0.60 ± 0.18	0.64 ± 0.21
Benzo[a]anthracene	0.04 ± 0.01	0.04 ± 0.03	0.07 ± 0.02	0.12 ± 0.002	5.98 ± 0.08	6.23 ± 0.13	6.73 ± 1.11
Chrysene	0.07 ± 0.02	0.06 ± 0.03	0.06 ± 0.04	0.06 ± 0.002	2.77 ± 0.08	2.93 ± 0.12	4.27 ± 2.13
Benzo[b]fluoranthene	3.38 ± 0.33	1.82 ± 0.05	2.05 ± 0.12	0.19 ± 0.0.01	9.41 ± 0.54	8.60 ± 0.09	9.85 ± 2.95
Benzo[k]fluoranthene	0.30 ± 0.04	0.08 ± 0.06	0.05 ± 0.07	0.002± 0.001	0.10 ± 0.07	0.18 ± 0.03	1.24 ± 1.95
Benzo[e]pyrene	0.28 ± 0.07	0.20 ± 0.09	0.03 ± 0.02	0.003 ± 0.002	0.13 ± 0.10	0.34 ± 0.11	1.68 ± 3.04
Perylene	1.93 ± 0.76	1.12 ± 0.14	0.10 ± 0.32	0.25 ± 0.03	12.73 ± 1.29	4.56 ± 0.55	5.12 ± 1.27
Indeno[123cd]pyrene	0.73 ± 0.05	0.71 ± 0.16	2.18 ± 2.23	nd	nd	0.39 ± 0.09	2.94 ± 4.69
Dibenz[a,h]anthracene	0.55 ± 0.56	0.20 ± 0.19	1.11 ± 1.69	nd	nd	30.96 ± 19.01	nd
Benzo[ghi]perylene	0.37 ± 0.32	0.32 ± 0.39	0.21 ±0.33	0.10 ± 0.01	4.91 ± 0.29	4.88 ± 0.08	6.31 ± 2.82

nd = not detected

Table S7. Blank concentrations (average ± standard deviation) of PCBs in field and method blanks. Field blank concentrations in LDPE passive samplers and XAD -air samplers are expressed both as ng/g and ng/ml for comparability with method blanks.

	LDPE – passive sampling polymer			XAD - air			Sediment and particle trap
	Field blank		method blank	Field blank		method blank	method blank
	ng/g	ng/ml	ng/ml	ng/g	ng/ml	ng/ml	ng/ml
PCB3	0.03 ± 0.02	0.02 ± 0.01	0.009 ± 0.006	nd	nd	0.008 ± 0.008	0.16 ± 0.12
PCB4	0.28 ±0.12	0.21 ± 0.15	0.008 ± 0.005	0.0005 ± 0.0004	0.03 ± 0.02	0.003 ± 0.001	0.15 ± 0.12
PCB18	0.01 ± 0.01	0.01 ± 0.01	0.009 ± 0.01	0.0008 ± 0.0002	0.04 ± 0.01	0.01 ± 0.005	0.05 ± 0.02
PCB20	0.10 ± 0.09	0.08 ± 0.1	nd	0.003 ± 0.0006	0.14 ± 0.03	0.03 ± 0.02	0.38 ± 0.18
PCB28	0.07 ± 0.04	0.04 ± 0.02	0.02 ± 0.01	0.005 ± 0.001	0.22 ± 0.07	0.07 ± 0.05	0.45 ± 0.25
PCB40	nd	nd	nd	0.01 ± 0.005	0.68 ± 0.25	0.37 ± 0.58	0.07 ± 0.03
PCB52	0.05 ± 0.04	0.03 ± 0.02	0.02 ± 0.02	0.002 ± 0.001	0.11 ± 0.06	0.07 ± 0.05	0.10 ± 0.03
PCB53	nd	nd	nd	nd	nd	nd	0.05 ± 0.02
PCB70	0.02 ± 0.01	0.01 ± 0.01	0.02 ± 0.006	0.001 ± 0.0005	0.05 ± 0.03	0.03 ± 0.02	nd
PCB101	0.02 ± 0.02	0.01 ± 0.01	0.01 ± 0.006	0.0005 ± 0.0002	0.02 ± 0.01	0.03 ± 0.03	0.10 ± 0.06
PCB110	0.02 ± 0.01	0.01 ± 0.01	0.01 ± 0.01	0.0007 ± 0.0004	0.03 ± 0.02	0.01 ± 0.01	0.04 ± 0.02
PCB118	0.01 ± 0.01	0.01 ± 0.004	0.006 ± 0.004	0.002 ± 0.00003	0.08 ± 0.02	0.02 ± 0.03	0.05 ± 0.03
PCB128	nd	nd	nd	nd	nd	nd	0.03 ± 0.01
PCB136	0.03 ± 0.02	0.02 ± 0.01	0.004 ± 0.002	0.0004 ± 0.0002	0.02 ± 0.01	0.01 ± 0.01	0.02 ± 0.01
PCB138	0.02 ± 0.01	0.02 ± 0.01	0.02 ± 0.01	0.002 ± 0.002	0.11 ± 0.08	0.05 ± 0.02	0.03 ± 0.01
PCB149	0.02 ± 0.01	0.01 ± 0.01	0.009 ± 0.006	0.0005 ± 0.0004	0.03 ± 0.02	0.01 ± 0.01	0.04 ± 0.03
PCB153	0.02 ± 0.01	0.01 ± 0.005	0.01 ± 0.01	0.0005 ± 0.0004	0.03 ± 0.02	0.02 ± 0.02	0.05 ± 0.03
PCB170	nd	nd	nd	nd	0.01 ± 0.01	0.01 ± 0.01	0.03 ± 0.01
PCB180	0.13 ± 0.29	0.08 ± 0.18	0.03 ± 0.03	0.001 ± 0.001	0.06 ± 0.05	0.01 ± 0.01	0.04 ± 0.02
PCB187	0.004 ± 0.004	0.003 ± 0.002	0.005 ± 0.003	0.003 ± 0.003	0.02 ± 0.02	0.01 ± 0.01	0.03 ± 0.01
PCB200	nd	nd	nd	0.0005 ± 0.0006	0.02 ± 0.03	0.01 ± 0.01	0.03 ± 0.01

nd = not detected

Table S8. Recoveries of internal surrogate standards (average ± standard deviation) in the samples. The data for D8-naphtalene in the particle trap samples, D8-acenapthylene in particle trap- and air samples, and D12-benzo(a)pyrene and C13-PCB 52 in air samples were excluded from the data set due to the low recoveries (highlighted in red in the table).

	LDPE	Soxhlet extraction		
	Passive sampling polymer	Sediment	Particle traps	XAD-air
D8-Naphthalene	64 ± 31%	31 ± 4%	*7 ± 0.4%*	25 ± 19%
D8-Acenaphthylene	54 ± 9%	47 ± 6%	*15 ± 4%*	*18 ± 8%*
D10-Fluorene	51 ± 8%	88 ± 29%	27 ± 5%	24 ± 11%
D10-Phenanthrene	56 ± 12%	75 ± 8%	48 ± 9%	33 ± 15%
D10-Anthracene	67 ± 15%	106 ± 4%	62 ± 21%	35 ± 26%
D10-Fluoranthene	90 ± 39%	96 ± 28%	35 ± 10%	62 ± 28%
D10-Pyrene	86 ± 34%	123 ± 7%	79 ± 16%	58 ± 25%
D12-Benzo[a]anthracene	122 ± 46%	74 ± 0.2%	48 ± 8%	45 ± 18 %
D12-Chrysene	93 ± 37%	63 ± 3%	46 ± 7%	45 ± 15%
D12-Benzo[a]pyrene	71 ± 17%	59 ± 6%	35 ± 9%	*17 ± 13%*
C13-PCB 28	69 ± 8%	78 ± 9%	54 ± 8%	37 ± 13%
C13-PCB 52	62 ± 10%	59 ± 10%	45 ± 7%	*20 ± 7%*
C13-PCB 101	80 ± 13%	85 ± 15%	49 ± 12%	34 ± 10%
C13-PCB 118	84 ± 13%	94 ± 17%	45 ± 9%	31 ± 10%
C13-PCB 138	87 ± 15%	83 ± 15%	41 ± 9%	38 ± 12%
C13-PCB 153	86 ± 15%	105 ± 15%	47 ± 9%	42 ± 15%
C13-PCB 180	95 ± 16%	124 ± 15%	54 ± 13%	43 ± 13%

Table S9. Fraction of PRCs left in the samplers after deployment (%) at the two sampling sites (Marine-OC and Terrestrial-OC) in surface water (W_S), the water column (W_M), bottom water (W_B) and sediment (Sed). Fractions close to (±5%), but beyond 20-80% are highlighted in grey, < 15% in light red and > 85% red. Red font-color indicates that the PRC dissipation was nominal (less than blank variation), 100% PRC was retained after sampling, and 0.01% dissipation was used in the calculations. For these compounds specifically, there is a risk that the calculated environmental concentrations are underestimated. e = equilibrium between the environment and sampler was reached.

	Marine-OC				Terrestrial-OC			
	W_S	W_M	W_B	Sed	W_S	W_M	W_B	Sed
13C-Phenanthrene	e	e	e	26%	e	e	E	*18%*
13C-Pyrene	e	*4%*	27%	73%	*17%*	e	E	74%
13C- Benzo[a]pyrene	46%	72%	***99.9%***	***99.9%***	38%	71%	***96%***	***99.9%***
C13-PCB8	e	*2%*	*4%*	43%	e	e	*3%*	43%
C13-PCB32	*4%*	*6%*	26%	77%	*3%*	*4%*	*5%*	58%
C13-PCB47	e	*15%*	58%	*83%*	e	e	47%	***90%***
C13-PCB111	*16%*	44%	***87%***	*85%*	*18%*	30%	77%	***95%***
PCB155	40%	61%	*84%*	***88%***	35%	54%	*83%*	***99.9%***

Table S10. Freely dissolved concentrations in surface water (C_{sw}), bottom water (C_{bw}), and surface sediment pore water (C_{pw}), and C_{pw}/C_{bw} ratio. OC-normalized concentrations in surface sediment (C_{sed}) and suspended particles from particle traps (C_{pt}), and air concentrations (C_{air}) at the sampling sites Terrestrial-OC and Marine-OC. Flux rates between sediment and water, and air and water (ng m^{-2} day^{-1} PAHs, pg m^{-2} day^{-1} PCBs) where positive values mean flux direction from sediment-to-water or water-to-air. The sediment-water interphase was considered to be at thermodynamic equilibrium (e) if the difference in concentration between phases was less than × 2, nd=not detected (not found or below LOD), -- data removed due to low recovery of the internal surrogate standards.

Terrestrial-OC

	C_{sw} ng l^{-1}	C_{bw} ng l^{-1}	C_{pw} ng l^{-1}	C_{pw}/C_{bw}	C_{sed} mg kg_{OC}^{-1}	C_{pt} mg kg_{OC}^{-1}	C_{air} ng m^{-3}	$Flux_{sed\_wat}$ ng m_2^{-1} day^{-1}	$Flux_{wat\_air}$ ng m_2^{-1} day^{-1}
Naphtalene	nd	8.94 ± 5.02	3.24	0.36	0.11 ± 0.01	--	1.19 ± 0.83	-311.53	
Acenapthene	0.39 ± 0.08	0.09 ± 0.03	0.07	0.75	0.02 ± 0.04	0.02 ± 0.01	nd	e	
Acenapthylene	nd	0.60 ± 0.12	0.56	0.94	0.02 ± 0.002	--	--	e	
Fluorene	0.42 ± 0.08	0.43 ± 0.06	0.32	0.75	0.04 ± 0.01	0.03 ± 0.01	0.15 ± 0.05	e	-3.79
Anthracene	0.02 ± 0.01	0.04 ± 0.02	0.04	1.23	0.10 ± 0.01	0.05 ± 0.02	nd	e	
Fluoranthene	0.10 ± 0.005	0.50 ± 0.02	0.61	1.22	0.75 ± 0.002	0.33 ± 0.05	0.06 ± 0.001	e	-40.45
Benzo[c]phenanthrene	0.25± 0.14	0.26 ± 0.02	0.54	2.03	0.14 ± 0.01	0.05 ± 0.005	nd	9.92	
Benzo[a]anthracene	0.004 ± 0.0002	0.003 ± 0.001	0.01	4.21	0.42 ± 0.02	0.14 ±0.03	nd	0.37	
Chrysene	0.02 ± 0.001	0.02 ± 0.003	0.05	2.26	0.61 ± 0.02	0.26 ± 0.02	0.002 ± 0.0002	1.10	-3.95
Benzo[b]fluoranthene	0.01 ± 0.002	0.12 ± 0.04	0.20	1.75	1.69 ± 0.02	0.83 ± 0.20	nd	e	
Benzo[k]fluoranthene	0.01 ± 0.001	0.03 ± 0.02	0.03	9.67	0.46 ± 0.04	0.28 ± 0.05	nd	1.01	
Benzo[e]pyrene	0.01 ±0.001	0.01 ± 0.004	0.05	3.90	0.92 ± 0.001	0.47 ± 0.07	nd	1.35	
Perylene	0.004 ± 0.001	0.01 ± 0.01	0.01	1.40	0.52 ± 0.004	0.23 ± 0.03	nd	e	
Indeno[123cd]pyrene	0.01 ± 0.002	0.01 ± 0.01	0.02	1.83	1.45± 0.1	0.67 ± 0.02	nd	e	
Benzo[ghi]perylene	0.002 ± 0.001	0.02 ± 0.01	0.02	0.91	1.84 ± 0.09	0.55 ± 0.02	nd	e	

Marine-OC

	C_{sw} ng l^{-1}	C_{bw} ng l^{-1}	C_{pw} ng l^{-1}	C_{pw}/C_{bw}	C_{sed} mg kg_{OC}^{-1}	C_{pt} mg kg_{OC}^{-1}	C_{air} ng m^{-3}	$Flux_{sed\_wat}$ ng m_2^{-1} day^{-1}	$Flux_{wat\_air}$ ng m_2^{-1} day^{-1}
Naphtalene	nd	3.73 ± 2.23	3.74	1.00	0.18 ± 0.02	--	1.59 ± 1.20	e	
Acenapthene	0.54 ± 0.08	0.07 ± 0.01	0.26	3.78	0.04 ± 0.002	0.01 ± 0.003	nd	9.14	
Acenapthylene	nd	0.44 ± 0.15	0.49	1.12	0.02 ± 0.002	--	--	e	
Fluorene	0.51 ± 0.01	0.56 ± 0.03	0.75	1.35	0.06 ± 0.005	0.02 ± 0.001	0.26 ± 0.06	e	-6.74
Anthracene	0.01 ± 0.01	0.02 ± 0.02	0.73	32.37	0.08 ± 0.01	0.03 ± 0.002	nd	30.56	
Fluoranthene	0.22 ± 0.005	0.81 ± 0.05	1.71	2.13	0.70 ± 0.03	0.26 ± 0.02	0.09 ± 0.01	35.93	-63.87
Benzo[c]phenanthrene	0.16 ± 0.09	0.32 ± 0.10	1.29	4.02	0.11 ± 0.02	0.03 ± 0.001	nd	35.04	
Benzo[a]anthracene	0.0007 ± 0.0002	0.003 ± 0.003	0.02	5.83	0.37 ± 0.002	0.08 ± 0.002	nd	0.50	
Chrysene	0.01 ± 0.0002	0.02 ± 0.01	0.08	3.11	0.58 ± 0.01	0.18 ± 0.01	0.003 ± 0.0002	1.90	-5.94
Benzo[b]fluoranthene	0.01 ± 0.001	0.05 ± 0.04	0.07	1.54	1.67 ± 0.10	0.42 ± 0.001	0.04 ± 0.05	e	
Benzo[k]fluoranthene	0.003 ± 0.0001	0.01 ± 0.01	0.01	1.63	0.43 ± 0.004	0.14 ± 0.02	0.004 ± 0.003	e	
Benzo[e]pyrene	0.004 ± 0.0002	0.01 ± 0.01	0.02	1.95	0.85 ± 0.02	0.24 ± 0.0003	nd	e	
Perylene	0.001 ± 0.0001	0.01 ± 0.01	0.01	1.80	0.31 ± 0.02	0.11 ± 0.01	nd	e	
Indeno[123cd]pyrene	0.003 ± 0.0001	0.003 ± 0.004	0.01	2.32	1.46 ± 0.13	0.45 ± 0.03	nd	0.11	
Benzo[ghi]perylene	0.001 ± 0.00005	0.01 ± 0.01	0.01	2.25	1.75 ± 0.15	0.39 ± 0.01	nd	0.21	

not detected in any samples: Dibenzothiophene, Diebenz[ah]anthracene

Terrestrial-OC

	C_{sw} pg l^{-1}	C_{bw} pg l^{-1}	C_{pw} pg l^{-1}	C_{pw}/C_{bw}	C_{sed} µg kg_{OC} -1	C_{pt} µg kg_{OC}^{-1}	$C_{air\ p}$g m^{-3}	$Flux_{sed\_wat}$ pg m_2^{-1} day^{-1}	$Flux_{wat\_air}$ pg m_2^{-1} day^{-1}
PCB4	36.31 ± 7.41	nd	nd		0.61 ± 0.21	6.24 ± 6.66	1.03 ± 0.21		2.04
PCB18	0.20 ± 0.08	nd	nd		0.26 ± 0.09	0.95	4.41 ± 0.23		-18.16
PCB20	nd	nd	nd		6.10 ± 0.42	18.75 ± 1.77	1.19 ± 0.17		
PCB28	nd	1.02 ± 0.25	1.71	1.67	3.71 ± 0.59	14.75 ± 5.35	6.48 ± 0.28	e	
PCB52	0.38 ± 0.10	0.66 ± 0.43	0.73	1.10	1.97 ± 0.47	1.59 ± 0.40	--		
PCB101	0.53 ± 0.05	0.69 ± 0.13	2.53	3.67	3.15 ± 0.02	2.30 ± 0.21	1.21 ± 0.11	51.85	-1.62
PCB110	0.16 ± 0.04	0.33 ± 0.11	nd		1.33 ± 0.003	1.64 ± 0.52	1.07± 0.10		-1.27
PCB118	0.14 ± 0.02	nd	nd		3.00 ± 0.19	2.21 ± 0.53	0.72 ± 0.21		-0.83
PCB128	nd	nd	nd		0.93 ± 0.30	0.49 ± 0.06	1.80 ± 2.71		
PCB136	nd	nd	nd		0.49 ± 0.03	0.26 ± 0.02	nd		
PCB138	0.30 ± 0.08	0.20 ± 0.17	2.47	12.37	8.56 ± 0.15	5.96 ± 0.23	nd	59.60	
PCB149	0.28 ± 0.02	0.21 ± 0.05	2.56	12.37	3.43 ±0.46	2.60 ± 0.56	1.04 ± 0.18	61.73	-0.35
PCB153	0.40 ± 0.04	0.36 ± 0.15	3.09	8.57	12.94 ± 0.64	10.20 ± 0.10	1.39 ± 0.14	71.43	-0.44
PCB180	0.05 ± 0.05	nd	nd		5.76 ± 0.26	6.23 ± 3.34	nd		
PCB187	0.03 ± 0.004	0.02 ± 0.01	0.14	9.00		1.46± 0.70	nd	3.05	

Marine-OC

	Cs_w pg l^{-1}	C_{bw} pg l^{-1}	C_{pw} pg l^{-1}	C_{pw}/C_{bw}	C_{sed} µg kg_{OC}^{-1}	C_{pt} µg kg_{OC}^{-1}	$C_{air\ p}$g m^{-3}	$Flux_{sed\_wat}$ pg m_2^{-1} day^{-1}	$Flux_{wat\_air}$ pg m_2^{-1} day^{-1}
PCB4	51.02 ± 4.38	nd	nd		1.19 ± 0.10	1.47 ± 0.92	1.61 ± 0.52		2.28
PCB18	nd	nd	nd		0.23 ± 0.04	3.31 ± 1.09	4.01 ± 0.63		
PCB20	nd	nd	nd		4.57 ± 0.50	39.07 ±47.66	0.81 ± 0.18		
PCB28	1.48 ± 0.22	1.43 ± 0.37	5.14	3.59	3.45 ± 0.02	9.26 ± 6.23	5.63 ± 0.20	123.40	-11.59
PCB52	0.36 ± 0.07	0.39 ± 0.35	1.25	3.20	2.47 ± 0.21	1.69 ± 0.34	--		
PCB101	0.23 ± 0.03	0.72 ± 0.49	0.51	0.70	3.49 ± 0.28	2.30 ± 0.07	1.32 ± 0.07	e	-1.79
PCB110	0.18 ± 0.02	0.51 ±0.21	0.70	1.38	2.31 ± 0.34	1.89 ± 0.22	0.74 ± 0.15	e	-0.87
PCB118	0.08 ± 0.01	nd	nd		3.63 ± 0.39	2.86 ± 0.67	0.42 ± 0.44		-0.49
PCB128	nd	nd	nd		1.14 ± 0.22	0.70 ± 0.45	0.16 ± 0.04		
PCB136	nd	nd	nd		0.52 ± 0.09	0.28 ± 0.01	0.15 ± 0.01		
PCB138	0.18 ± 0.06	0.20 ± 0.10	0.50	2.49	9.18 ± 1.21	7.12 ± 0.39	nd	7.82	
PCB149	0.15 ± 0.04	0.21 ± 0.14	0.43	2.05	3.53 ± 0.38	2.46 ± 0.16	0.68 ± 0.20	5.82	-0.23
PCB153	0.24 ± 0.02	0.39 ± 0.32	0.32	0.81	12.19 ± 0.15	9.46 ± 3.83	1.12 ± 0.23	e	-0.36
PCB180	0.04 ± 0.04	nd	nd		6.56 ± 0.21	9.77 ± 4.47	nd		
PCB187	0.02 ± 0.01	0.04 ± 0.02	0.02	0.55	1.27 ± 0.35	1.04 ± 0.22	nd	e	

not detected in any samples: PCB3, PCB40, PCB53, PCB 70, PCB200

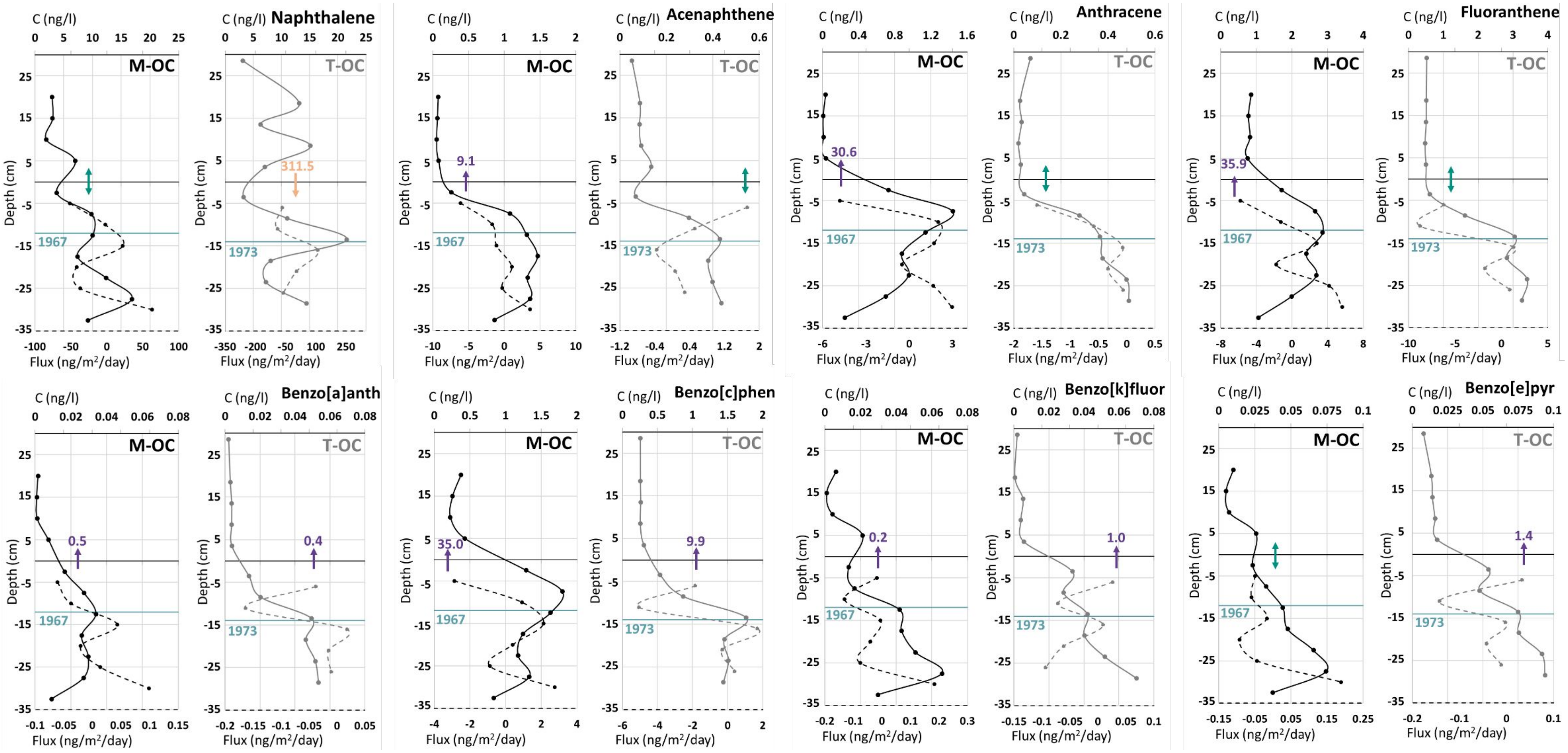

Figure S5. Sediment pore-water concentrations, and bottom water concentrations of selected PAHs (ng l^{-1}, solid line) at different depths (cm), and the diffusive flux rates (ng m^{-2} day^{-1}) within the sediment and at the sediment-water interphase (dotted line) at the two sites: Marine-OC (**M-OC**) and Terrestrial-OC (**T-OC**). Flux direction at the sediment-water interphase is expressed with arrows. Sediment dating at 1967 (M-OC) and 1973 (T-OC) indicated with a horizontal line. The data is expressed with two horizontal axes.

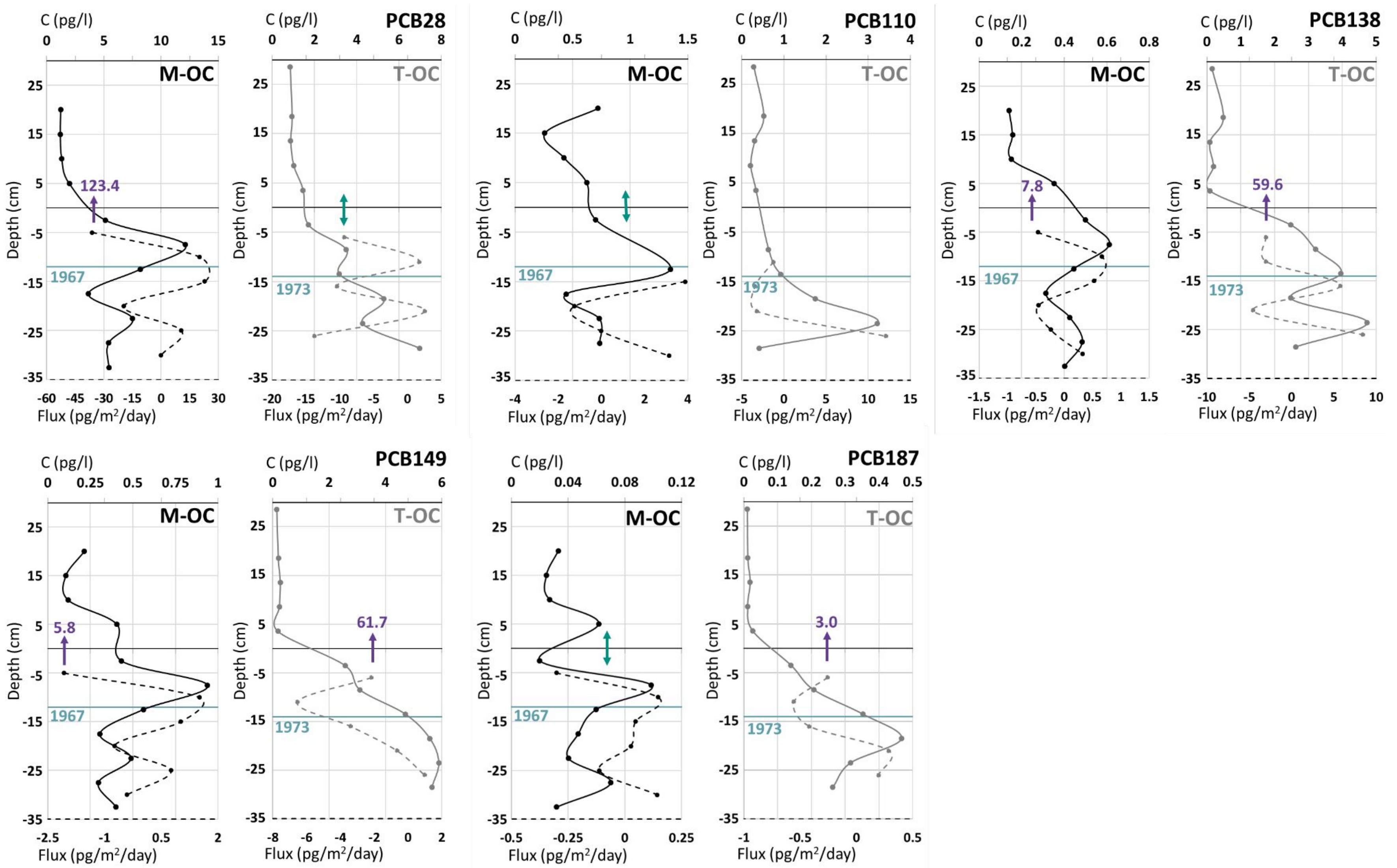

Figure S6. Sediment pore-water concentrations, and bottom water concentrations of PCBs (pg l^{-1}, solid line) at different depths (cm), and the diffusive flux rates (pg m^{-2} day^{-1}) within the sediment and at the sediment-water interphase (dotted line) at the two sites: Marine-OC (**M-OC**) and Terrestrial-OC (**T-OC**). Flux direction at the sediment-water interphase is expressed with arrows. Sediment dating at 1967 (M-OC) and 1973 (T-OC) indicated with a horizontal line. The data is expressed with two horizontal axes.

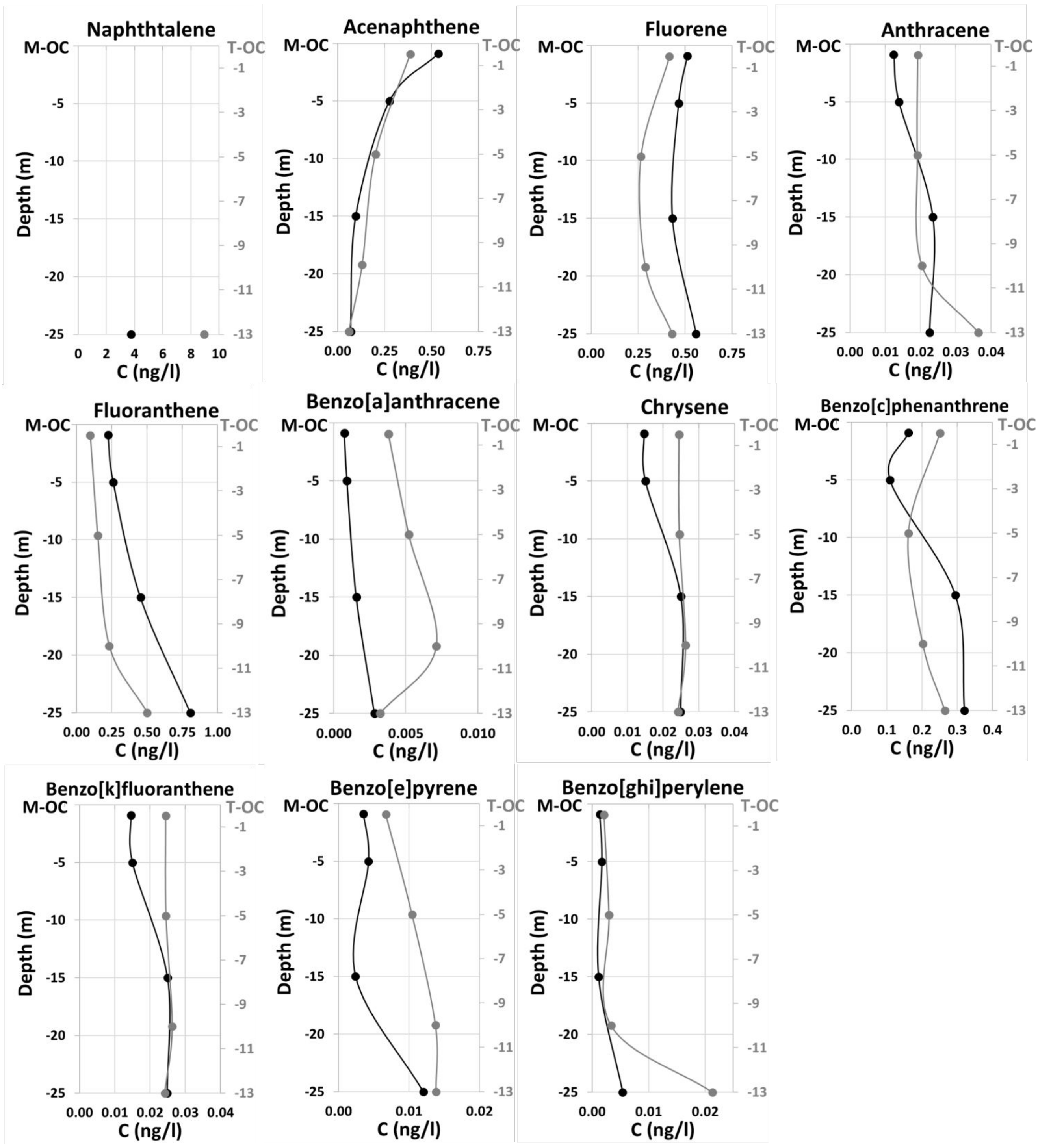

Figure S7. Water concentrations (ng l^{-1}) of selected PAHs at different water depths. The data is expressed with two vertical axes for the different water depths at the study sites: Marine-OC (**M-OC**) on the left (0-25 m, black) and Terrestrial-OC (**T-OC**) on the right (0-13m, grey). Naphthalene was only observed in the bottom water near the sediment surface.

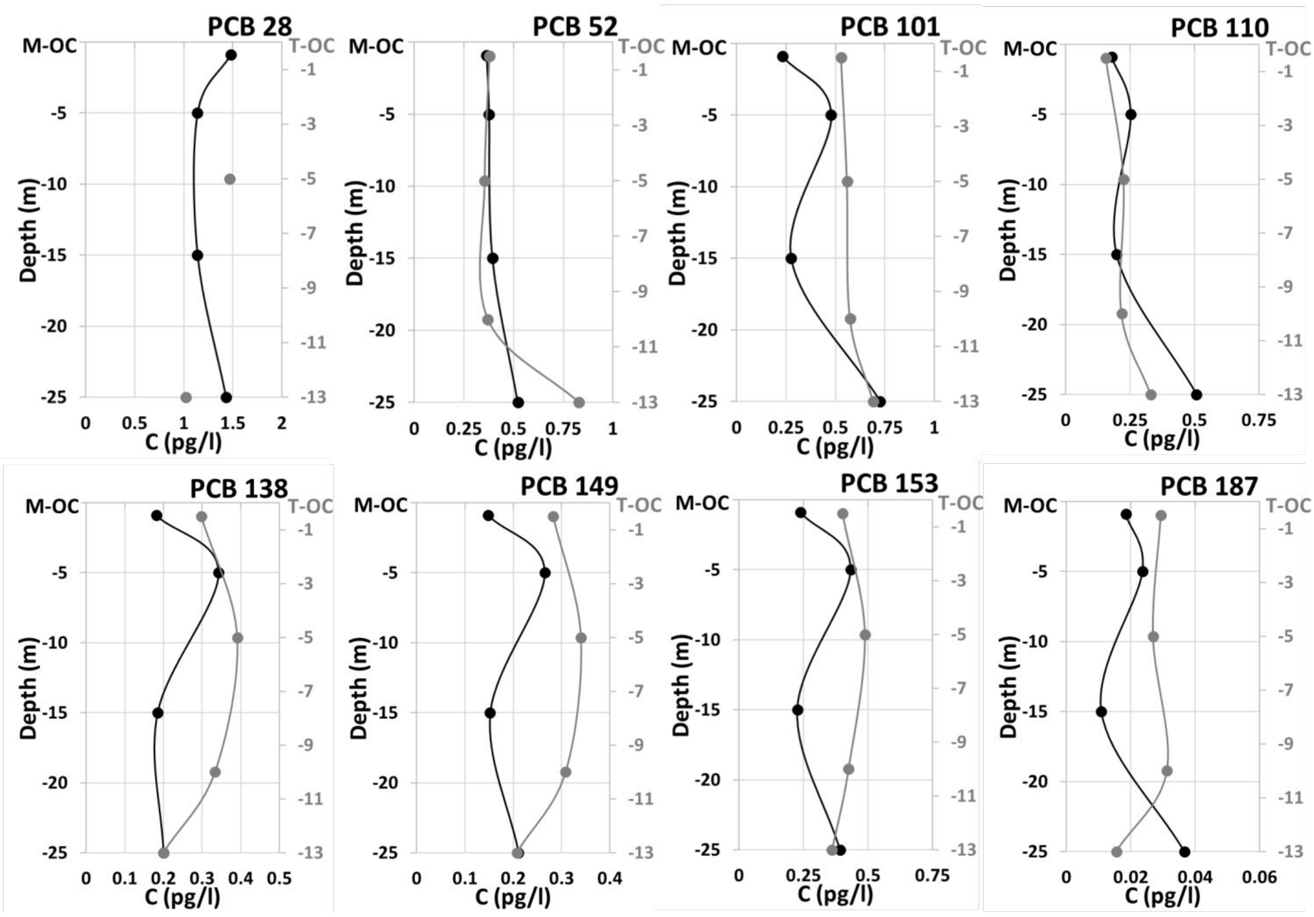

Figure S8. Water concentrations (pg l^{-1}) of PCBs at different water depths. The data is expressed with two vertical axes for the different water depths at the study sites: Marine-OC (**M-OC**) on the left (0-25 m, black) and Terrestrial-OC (**T-OC**) on the right (0-13m, grey). At the Terrestrial-OC site PCB 28 was observed only at two water depths (13m and 5m).

Table S11. Site-specific OC-water partition coefficients, log $K_{oc(obs)}$ at both sites: Terrestrial-OC and Marine-OC. Log $K_{oc(obs)}$ calculated as a ratio of the organic carbon normalized sediment concentrations, and the sediment pore-water concentrations in the surface sediment (sediment), and organic carbon normalized concentration in suspended particles and water concentration at the deployment depth of the particle traps (particle trap).

	**Terrestrial-OC**		**Marine-OC**	
	Log $K_{oc(obs)}$ sediment	Log $K_{oc(obs)}$ particle trap	Log $K_{oc(obs)}$ sediment	Log $K_{oc(obs)}$ particle trap
Naphtalene	4.54		4.67	
Acenapthene	5.54	4.74	5.15	
Acenapthylene	4.62		4.54	
Fluorene	5.05	4.92	4.91	4.71
Anthracene	6.37	6.17	5.21	6.08
Fluoranthene	6.09	6.24	5.61	5.75
Benzo[a]anthracene	7.49	7.21	7.35	7.73
Chrysene	7.05	6.85	6.87	6.85
Benzo[b]fluoranthene	6.92	7.14	7.38	7.95
Benzo[k]fluoranthene	7.14	7.23	7.51	7.98
Benzo[e]pyrene	7.23	7.37	7.56	8.01
Indeno[123cd]pyrene	7.84	7.71	8.36	8.54
Benzo[ghi]perylene	7.98	8.12	8.16	8.54

	Log $K_{oc(obs)}$ sediment	Log $K_{oc(obs)}$ particle trap	Log $K_{oc(obs)}$ sediment	Log $K_{oc(obs)}$ particle trap
PCB4		5.76		4.92
PCB18	5.79	6.59	4.93	
PCB28	6.34	7.18	5.83	6.91
PCB52	6.43	6.65	6.29	6.63
PCB101	6.09	6.61	6.84	6.92
PCB110		6.86	6.52	6.98
PCB118		7.09		7.85
PCB136	6.14			
PCB138	6.54	7.18	7.26	7.58
PCB149	6.13	6.88	6.91	7.21
PCB153	6.62	7.32	7.58	7.62
PCB180		7.88		8.40
PCB187	6.97	7.74	7.80	7.98

References

(1) Birch, H.; Gouliarmou, V.; Lutzhoft, H. H.; Mikkelsen, P. S.; Mayer, P. Passive Dosing to Determine the Speciation of Hydrophobic Organic Chemicals in Aqueous Samples. *Anal. Chem.* **2010,** *82* (3), 1142-1146.

(2) Mandalakis, M.; Zebuhr, Y.; Gustafsson, Ö. Efficient isolation of polyaromatic fraction from aliphatic compounds in complex extracts using dimethylformamide-pentane partitionings. *Journal of Chromatography a* **2004,** *1041* (1-2), 111-117.

(3) Mustajärvi, L.; Eek, E.; Cornelissen, G.; Eriksson-Wiklund, A.; Undeman, E.; Sobek, A. In situ benthic flow-through chambers to determine sediment-to-water fluxes of legacy hydrophobic organic contaminants. *Environmental Pollution* **2017,** *231* 854-862.

(4) Nybom, I.; Waissi-Leinonen, G.; Mäenpää, K.; Leppänen, M. T.; Kukkonen, J. V. K.; Werner, D.; Akkanen, J. Effects of activated carbon ageing in three PCB contaminated sediments: Sorption efficiency and secondary effects on *Lumbriculus variegatus*. *Water Res.* **2015,** *85* 413-421.

(5) Booij, K.; Sleiderink, H.; Smedes, F. Calibrating the uptake kinetics of semipermeable membrane devices using exposure standards. *Environmental Toxicology and Chemistry* **1998,** *17* (7), 1236-1245.

(6) Bartkow, M. E.; Jones, K. C.; Kennedy, K. E.; Holling, N.; Hawker, D. W.; Mueller, J. F. Evaluation of performance reference compounds in polyethylene-based passive air samplers. *Environmental Pollution* **2006,** *144* (2), 365-370.

(7) Lohmann, R. Critical Review of Low-Density Polyethylene's Partitioning and Diffusion Coefficients for Trace Organic Contaminants and Implications for Its Use As a Passive Sampler. *Environ. Sci. Technol.* **2012,** *46* (2), 606-618.

(8) Schwarzenbach, R.P.; Gschwend, P.M.; Imboden, D.M. *Environmental Organic Chemistry.* Wiley Interscience: New York, 2003;

(9) Boudreau, B. P. The diffusive tortuosity of fine-grained unlithified sediments. *Geochim. Cosmochim. Acta* **1996,** *60* (16), 3139-3142.

(10) Boudreau, B.P. Solute transport above the sediment-water interface, In *The Benthic boundary layer Transport processes and biogeochemistry,* Boudreau, B.P. and Jørgensen, B.B., Eds.; Oxford University Press: UK, 2001; pp. 104-126.

(11) Eek, E.; Cornelissen, G.; Breedveld, G. D. Field Measurement of Diffusional Mass Transfer of HOCs at the Sediment-Water Interface. *Environ. Sci. Technol.* **2010,** *44* (17), 6752-6759.

(12) Wania, F.; Shen, L.; Lei, Y.; Teixeira, C.; Muir, D. Development and calibration of a resin-based passive sampling system for monitoring persistent organic pollutants in the atmosphere. *Environ. Sci. Technol.* **2003,** *37* (7), 1352-1359.

(13) Armitage, J. M.; Hayward, S. J.; Wania, F. Modeling the Uptake of Neutral Organic Chemicals on XAD Passive Air Samplers under Variable Temperatures, External Wind Speeds and Ambient Air Concentrations (PAS-SIM). *Environ. Sci. Technol.* **2013,** *47* (23), 13546-13554.

(14) Sabljic, A.; Gusten, H.; Hermens, J.; Opperhuizen, A. Modeling Octanol Water Partition-Coefficients by Molecular Topology - Chlorinated Benzenes and Biphenyls. *Environ. Sci. Technol.* **1993,** *27* (7), 1394-1402.

(15) Ma, Y.; Lei, Y. D.; Xiao, H.; Wania, F.; Wang, W. Critical Review and Recommended Values for the Physical-Chemical Property Data of 15 Polycyclic Aromatic Hydrocarbons at 25 degrees C. *J. Chem. Eng. Data* **2010,** *55* (2), 819-825.

(16) Bamford, H.; Poster, D.; Huie, R.; Baker, J. Using extrathermodynamic relationships to model the temperature dependence of Henry's law constants of 209 PCB congeners. *Environ. Sci. Technol.* **2002,** *36* (20), 4395-4402.

(17) Bamford, H.; Offenberg, J.; Larsen, R.; Ko, F.; Baker, J. Diffusive exchange of polycyclic aromatic hydrocarbons across the air-water interface of the Patapsco River, an urbanized subestuary of the Chesapeake Bay. *Environ. Sci. Technol.* **1999,** *33* (13), 2138-2144.

(18) Hornbuckle, K.; Jeremiason, J.; Sweet, C.; Eisenreich, S. Seasonal-Variations in Air-Water Exchange of Polychlorinated-Biphenyls in Lake-Superior. *Environ. Sci. Technol.* **1994,** *28* (8), 1491-1501.
